# CRISPR-powered optothermal nanotweezers: Diverse bio-nanoparticle manipulation and single nucleotide identification

**DOI:** 10.1038/s41377-023-01326-9

**Published:** 2023-11-16

**Authors:** Jiajie Chen, Zhi Chen, Changle Meng, Jianxing Zhou, Yuhang Peng, Xiaoqi Dai, Jingfeng Li, Yili Zhong, Xiaolin Chen, Wu Yuan, Ho-Pui Ho, Bruce Zhi Gao, Junle Qu, Xueji Zhang, Han Zhang, Yonghong Shao

**Affiliations:** 1 State Key Laboratory of Radio Frequency Heterogeneous Integration, Key Laboratory of Optoelectronic Devices and Systems of Ministry of Education and Guangdong Province, College of Physics and Optoelectronics Engineering, Shenzhen University, Shenzhen, 518060 China; 2 Department of Biomedical Engineering, The Chinese University of Hong Kong, Shatin, Hong Kong China; 3 Department of Bioengineering and COMSET, Clemson University, Clemson, SC 29634 USA; 4 School of Biomedical Engineering, Shenzhen University, Shenzhen, Guangdong 518060 China

**Keywords:** Optical manipulation and tweezers, Nanophotonics and plasmonics, Optical sensors

## Abstract

Optothermal nanotweezers have emerged as an innovative optical manipulation technique in the past decade, which revolutionized classical optical manipulation by efficiently capturing a broader range of nanoparticles. However, the optothermal temperature field was merely employed for in-situ manipulation of nanoparticles, its potential for identifying bio-nanoparticles remains largely untapped. Hence, based on the synergistic effect of optothermal manipulation and CRIPSR-based bio-detection, we developed CRISPR-powered optothermal nanotweezers (CRONT). Specifically, by harnessing diffusiophoresis and thermo-osmotic flows near the substrate upon optothermal excitation, we successfully trapped and enriched DNA functionalized gold nanoparticles, CRISPR-associated proteins, as well as DNA strands. Remarkably, we built an optothermal scheme for enhancing CRISPR-based single-nucleotide polymorphism (SNP) detection at single molecule level, while also introducing a novel CRISPR methodology for observing nucleotide cleavage. Therefore, this innovative approach has endowed optical tweezers with DNA identification ability in aqueous solution which was unattainable before. With its high specificity and feasibility for in-situ bio-nanoparticle manipulation and identification, CRONT will become a universal tool in point-of-care diagnosis, biophotonics, and bio-nanotechnology.

## Abstract (web summary)

We develop optothermal tweezers to boost CRISPR-based single-nucleotide polymorphism (SNP) detection at the single-molecule level. Additionally, this innovative approach has endowed optical tweezers with DNA identification ability.

## Graphical abstract

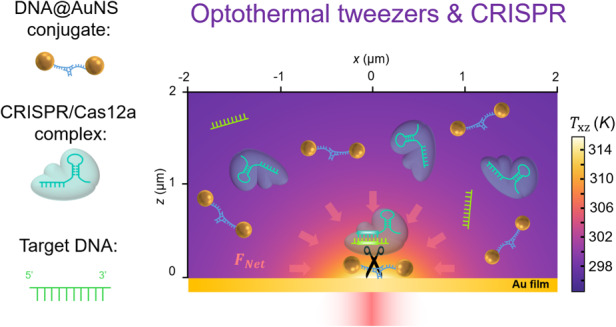

## Introduction

Since the invention of optical tweezers by Arthur Ashkin^1,2^, optical manipulation technique has become a powerful tool for the remote manipulation of nanoobjects, and it was awarded the Nobel Prize in Physics for its groundbreaking contributions to biological systems at 2018^1,3^. Classical optical tweezers are mainly depending on the momentum transform of the light, therefore, high numerical aperture (NA) objective lens and high laser power density is required, and diffraction of light also limits the manipulation accuracy. Fortunately, interdisciplinary combinations with fields such as plasmonic optics^4^, electrical field^5,6^, or temperature field^7–9^ have effectively addressed these limitations. As a result, various innovative approaches have emerged, offering new opportunities in particle analysis and manipulation. Remarkably, the optothermal nanotweezers, which utilize optical-induced thermodynamic forces, can manipulate nanoparticles in micrometer scale range with sub-wavelength precision^8^. Compared to traditional optical tweezers, the optothermal tweezers technique requires a rather lower laser power density (10–100 μW·μm^−2^), which makes them an attractive alternative for biological detection and reducing adverse optical effects on the biological samples subjected to manipulation. It can manipulate bio-nanoparticles ranging from micrometer to nanometer sizes, including bacteria and live cell^10–13^, as well as single- and double-strand DNA molecules (ss- and dsDNA)^14–16^, and proteins^17,18^. However, the in-situ biomolecule identification ability in liquid medium is yet a thorny issue for optical tweezers, which greatly hinders the technique to become a universal tool in point-of-care diagnosis and bio-nanotechnology. Furthermore, the temperature field in optothermal nanotweezers is presently limited to nanoparticle manipulation and remains largely untapped for its potential impact on biological reactions. Given that thermal effects play a crucial role in various biological processes, there exists an enormous opportunity to leverage the capabilities of the temperature field in optothermal nanotweezers for practical applications.

Notably, the clustered regularly interspaced short palindromic repeat (CRISPR) system is a remarkable gene editing tool and was also awarded Nobel prize in 2020^19^. It comprises of a CRISPR-associated (Cas) nuclease protein and a target DNA-specific guide RNA (crRNA). When activated by a matching target DNA (ssDNA or dsDNA) at certain temperature condition (~37 °C), cis- and trans-cleavage can be triggered. Therefore, it can be implemented as a target DNA detector with high recognition specificity at the single-base level^20,21^. Despite the high specificity of CRISPR-Cas systems, their sensitivity is often limited by the low abundance of target DNA in complex samples. For instance, CRISPR-Cas13 assays demonstrate >95% sensitivity and >99% specificity when detecting SARS-CoV-2 RNA, but only at concentrations above 10 copies per microliter^22^. Consequently, there is a need to improve the sensitivity of CRISPR-based detection methods, particularly for applications requiring early diagnosis at low-cost. One promising strategy involves combining advanced sensing techniques with CRISPR-Cas systems to amplify the signal or enrich the target DNA. For example, the UCAD (ultrasensitive CRISPR-based antibody detection) assay uses phase separation to increase the local concentration of fluorescent proteins, achieving attomolar sensitivity for antibody detection^23^. Another approach, CRISPR FISHer, exploits protein trimerization and phase separation to visualize nonrepetitive DNA sequences in living cells with high brightness and a strong signal-to-background ratio^24^. These methods highlight the potential of integrating CRISPR-Cas systems with novel sensing modalities to enhance the sensitivity and versatility of DNA detection. Nevertheless, the current methods still face challenges such as low multiplexing capability, high background noise, or limited applicability to different sample types.

Therefore, to break the limitations from both optothermal tweezers and CRISPR-based detection systems, we designed a universally applicable optothermal tweezing platform termed CRISPR-powered optothermal nanotweezers (CRONT). The CRONT can be exquisitely tuned to achieve bio-nanoparticles manipulation and meet the CRISPR working condition for target bio-nanoparticles identification. Specifically, by incorporating optothermal induced diffusiophoretic force^25,26^, we have successfully manipulated bio-nanoparticles of ssDNA, dsDNA, BSA, Cas12a protein and DNA-linked gold nanospheres (DNA@AuNS) conjugate. Subsequently, by incorporating a CRISPR-based DNA biosensing scheme, in which the cleavage of a single trapped DNA@AuNS conjugate is interrogated, we turned this optothermal tweezer into a molecular probe for in-situ DNA molecules (from SARS-CoV-2 or Monkeypox virus) identification without the need for nucleic acid amplification. And detection limits of 25 aM (ssDNA) and 250 aM (dsDNA) are achieved. Remarkably, our experiments have revealed that at ultra-lower detection volume (10 μL), this nanotweezers can also offer the ability to identify single nucleotide polymorphisms (SNPs), which play a crucial role in genetic diversity and are associated with various phenotypic traits, including disease susceptibility and drug response^27,28^. Therefore, this innovation in SNP detection techniques are essential to meet the diverse demands of genomic research and medicine in the future.

## Results

### Working principle and DNA@AuNS trapping

**Fig. 1:**
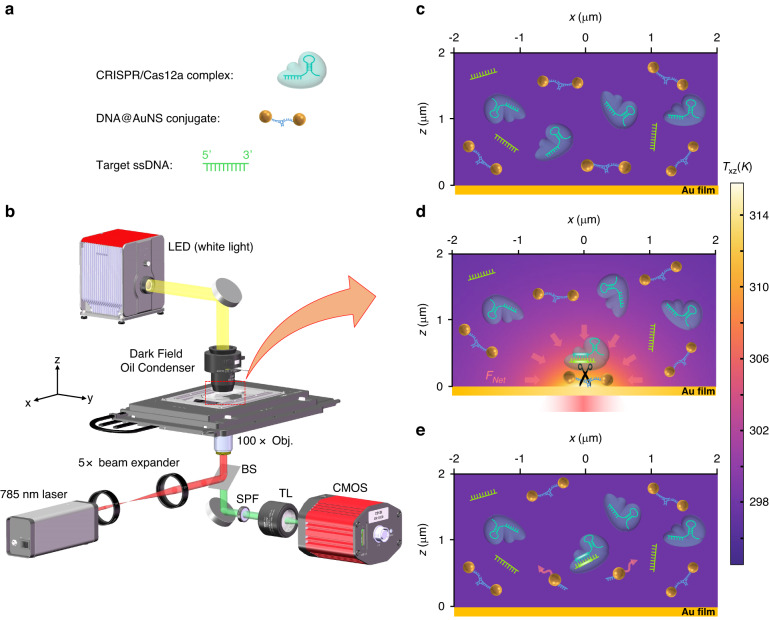
Working principle of CRONT. **a** The diagrammatic sketch of the three components in the solution: DNA@AuNS conjugate, CRISPR/Cas12a complex, and target ssDNA. **b** Optical setup, the BS, SPF, and TL are beam splitter, short pass filter, and tube lens (*f* = 200 mm), respectively. Additional details of the setup are provided in the Materials and Methods section. **c** Dispersion of the three components in the solution without optical heating. **d** Optothermal net force (${{\boldsymbol{F}}}_{{\boldsymbol{Net}}}$) induced migration and DNA@AuNS conjugate cleavage upon optical heating, the heating laser power is 0.5 mW. **e** Observation of the cleavage after the optical heating is switched off

To enable CRONT, we designed a microfluidic chamber incorporating a thin layer of gold (Au) film deposited on the bottom cover glass. Upon focused laser illumination, as shown in Fig. 1, the Au film (with an absorption rate of 40% at 785 nm) generates a temperature field surrounding the laser spot. At an optical power of 0.5 mW, the average temperature rise is 12 K (37 °C), which is optimal for CRISPR reactions (see temperature measurement method in Supplementary Note 1). Simultaneously, the thermodynamic net force ${{\boldsymbol{F}}}_{{\boldsymbol{Net}}}$ generated by optothermal tweezers gather the three CRISPR components (as shown in Fig. 1a) to this specific region. The CRISPR Cas12a protein then initiates cleavage of the DNA@AuNS conjugate (Fig. 1e), which is subsequently observed using dark-field microscopy. Specifically, a nonionic polymer of polyethylene glycol (PEG) with a molecular weight of ${M}_{w}=10000$ was added to the water solution, which is a widely used biological surfactant known for its excellent biocompatibility^29,30^. Our experiments are within the semidilute concentration regime of polymer (PEG-10k, wt>5%)^31^.

Under a temperature gradient (∇T), because of the thermophoresis, gold nanoparticle, polymer, protein or nucleic acid in the solution will move along the temperature gradient, and the drifting velocity is normally described by $v=-{D}_{T}\nabla T$, where ${D}_{T}$ is thermophoretic mobility of the particle, and a negative ${D}_{T}$ will drive the particle towards the hotter side, while a positive one will drive the particle towards the cold side. Therefore, the solute current is composed of a thermophoretic part and a diffusion part:

1 $${\boldsymbol{J}}=-n{D}_{T}\nabla T-D\nabla n$$

where *n* and *D* is the number of particles per unit volume and the Einstein diffusion coefficient. At dynamic equilibrium state, ***J*** = 0, considering one type of particle which ${D}_{T}$ and $D$ are constant, one can obtain the concentration distribution after integration, i.e.:

2 $$n\left(r\right)={n}_{0}\exp [-{S}_{T}\Delta T\left(r\right)]$$

where $\Delta T$ is the spatial temperature different and the Soret coefficient is ${S}_{T}={D}_{T}/D$.

When multiple nanoparticles coexist, their varying ${D}_{T}$ values can generate distinct solute concentration distributions in relation to the same ∇T. Solutes with higher concentrations can then influence solutes with lower concentrations via osmotic pressure, resulting in an interaction commonly known as diffusiophoretic force (${{\boldsymbol{F}}}_{{\boldsymbol{D}}}$)^25,26^. It can induce solute accumulations or separations, particularly for DNAs^14,16,32–34^. In our approach, the majority solute of PEG molecules, exhibit positive thermophoretic mobility. While the nanoparticles in the solution are the minority solutes, irrespective of their own thermophoretic behaviors (most bionanoparticle exhibit a positive ${D}_{T}$), as there exist enough amount of surrounding PEG molecules for particle-polymer boundary interactions, the diffusiophoretic force from PEG molecules can drive the nanoparticle towards the hotter region. In this scenario, the resultant transport velocity of the nanoparticle becomes:^25,35^

3 $$v=-({D}_{T}-{D}_{T}^{{diff}})\nabla T$$

Where ${D}_{T}^{{diff}}=\frac{{k}_{B}}{3\eta }{R}_{g}^{2}n\left(T{S}_{T}^{{PEG}}-1\right)$ is the diffusiophoresis term induced by majority solute of PEG^25^. The ${k}_{B}$, $\eta$,${S}_{T}^{{PEG}}$ and ${R}_{g}$ is Boltzmann constant, solution viscosity, Soret coefficient and gyration radius of PEG molecules, respectively. Therefore, for gold nanoparticles of diameters larger than the PEG network correlation length ξ (See Supplementary Note 2 for details), the ${D}_{T}-{D}_{T}^{{diff}}$ < 0, which means the nanoparticle can be driven to the laser heating center (The corresponding calculation is shown in Supplementary Note 3).

**Fig. 2:**
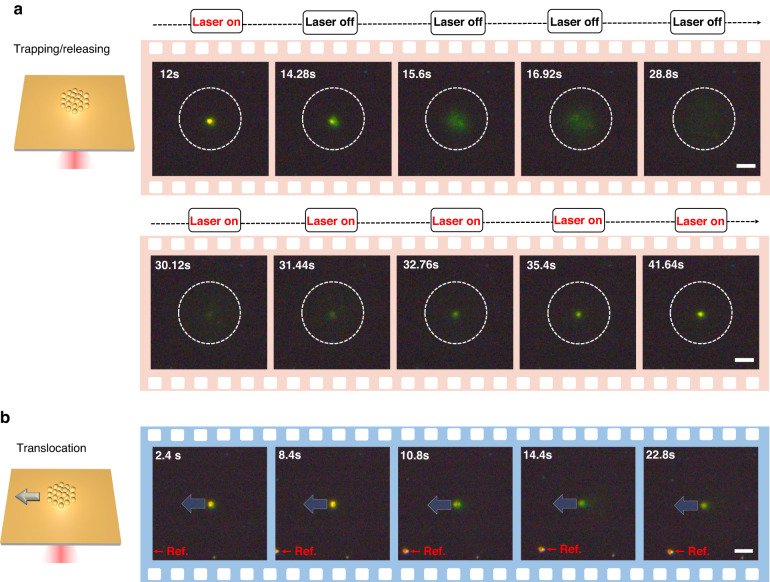
Successive microscope frames showing DNA@AuNS trapping, releasing, and translocation. **a** A frame-by-frame sequence of DNA@AuNS trapping and releasing, the dotted circle indicates the trapping region. **b** A frame-by-frame sequence of the translocation of a cluster of trapped DNA@AuNSs. The laser power is 0.33 mW, the AuNS and PEG concentration is 100 μM and 10% (wt%) respectively. Scaler bar = 4 μm. The full video can be accessed in Supplementary Video 1, 2

In addition, other nanoparticle-independent hydrodynamic phenomenon also coexists, our experiments were conducted in a rather shallower microfluidic chamber (height = 30 μm), at such height the buoyancy driven natural convective flow is suppressed due to the lower Rayleigh number^36,37^. However, the thermo-osmotic flow, i.e., another toroidal shape slip flow parallel to the solid–liquid interface^38^, also plays an important role in our trapping scheme. This is a kind of a micrometer-scale flow that arises from the $\nabla T$ induced excess enthalpy ${\boldsymbol{h}}$ at the interface between the Au-substrate and liquid. And the thermo-osmotic flow velocity ${v}_{{TO}}$ obeys:^38^

4 $${v}_{{TO}}\cdot \hat{{\boldsymbol{t}}}=\chi \frac{\nabla T}{T}\cdot \hat{{\boldsymbol{t}}}$$

where $\hat{{\boldsymbol{t}}}$, $\chi$ is the unit vector tangential to the substrate-liquid interface and thermo-osmotic coefficient, respectively. A positive $\chi$ means the liquid flows towards the hot side near the interface, while a negative one means the liquid flows towards the cold side. In our case, the thermo-osmotic coefficients of AuNS are χ_Au_ = 3.9 × 10^−10^ m^2^·s^−1^ (See Supplementary Note 4 for details). As shown in Fig. 2, we have trapped a cluster of DNA@AuNS to perform aggregation and translocation at the laser focusing center. Although it is commonly believed that the AuNS is hardly trapped via optothermal tweezers because of the larger thermal conductivity of metal nanoparticles, which leads to the surrounding temperature field homogenization^39^. Because of the DNA surface modification and the smaller size, the DNA@AuNS can still be steadily trapped (See Supplementary Note 4 for details).

**Fig. 3:**
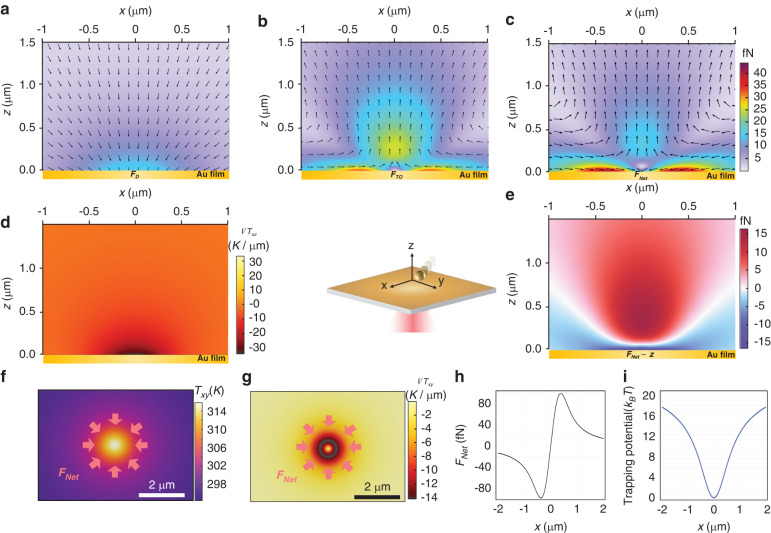
Force distribution of the CRONT in radial direction. Spatial force distributions in xz-plane. **a** Diffusiophoretic force (${{\boldsymbol{F}}}_{{\boldsymbol{D}}}$), **b** thermo-osmotic force (${{\boldsymbol{F}}}_{{\boldsymbol{TO}}}$), and **c** net force (${{\boldsymbol{F}}}_{{\boldsymbol{Net}}}$). **d** The temperature gradi**e**nt in xz-plane. **e** Force distribution of z-component of the net force (${{\boldsymbol{F}}}_{{\boldsymbol{Net}}{\boldsymbol{-}}{\boldsymbol{z}}}$). **f** The temperature distribution in xy-plane. **g** Temperature gradient distribution in xy-plane. **h** The magnitude of the net force distribution in 10 nm above the Au film, corresponding to a trapping stiffness of 272 fN·μm^−1^. **i** The trapping potential in terms of K_B_T. The heating laser spot center is located at (0, 0). The optical power of the 785 nm laser is 0.5 mW. The analyzed nanoparticle is a spherical DNA@AuNS conjugate with a diameter of 80 nm. Detail of temperature measurement and simulation are shown in the “Materials and methods” section

To assess the feasibility of this trapping scheme to perform CRONT, we analyzed the distribution of thermodynamic forces on a single DNA@AuNS at the optimized CRISPR temperature (37 °C). As illustrated in Fig. 3a–c, diffusiophoretic force (${{\boldsymbol{F}}}_{{\boldsymbol{D}}}$), thermo-osmotic force (${{\boldsymbol{F}}}_{{\boldsymbol{TO}}}$), and net force (${{\boldsymbol{F}}}_{{\boldsymbol{Net}}}$) are presented under $\nabla T$ of about 10 K·μm^−1^ (Fig. 3d). These thermodynamic forces are calculated via Stokes’ Law equation $F=6\pi \eta {Ru}$, after determining the values of $v$ and ${v}_{{TO}}$ from Eqs. 3 and 4, respectively. Here, R represents the radius of the target particle, $u$ denotes the relative velocity, and $\eta$ stands for the solution viscosity. In addition, we estimated the optical forces (${{\boldsymbol{F}}}_{{\boldsymbol{OPT}}}$) using the dipole approximation^40^, which is about one order of magnitude smaller than the dominated thermodynamic forces (***F***_***D***_, ***F***_***TO***_) in this trapping scheme (see Supplementary Note 5 for details). We also attempted to trap gold nanoparticles at an incident laser power of 0.5 mW without the deposition of a gold film, but it proved unattainable due to the insufficient power level.

In addition, as shown in Fig. 3e, because of the existence of the positive ${{\boldsymbol{F}}}_{{\boldsymbol{Net}}{\boldsymbol{-}}{\boldsymbol{z}}}$ near the trapping center, most of the particles are trapped from the radial direction and there is no particle captured from positive z-direction. Therefore, in CRONT, the thermo-osmotic flow will bring the nanoparticles from a radial distance of several micrometers away to the hotter center, and then diffusiophoretic force (${{\boldsymbol{F}}}_{{\boldsymbol{D}}}$) helps the nanoparticle to be tightly trapped at the heating center. The xy-plane force distribution and trapping potential were also analyzed (Fig. 3f–i), yielding a trapping stiffness of 269 fN·μm^−1^. Noted that in the simulation, we also accounted for the temperature homogeneity effect^39^ resulting from the combined thermal conductivity of gold and DNA (see Supplementary Note 6).

Furthermore, temperature fluctuations also impact these two thermal forces. In the case of the diffusiophoresis force, as indicated by Eq. 2, the PEG concentration term decreases exponentially with temperature $T$. Consequently, from Eq. 3, we can infer that ${D}_{T}^{{diff}}$ is positive and it decreases with temperature T. As a result, given that ${D}_{T}-{D}_{T}^{{diff}} < 0$, an increase in $T$ leads to a reduction in the absolute value of ${D}_{T}-{D}_{T}^{{diff}}$. Conversely, concerning the thermo-osmotic force, as expressed in Equation S1 from Supplementary Note 4, the thermo-osmotic coefficient χ becomes larger with increasing *T*. Therefore, a rise in temperature enhances the thermo-osmotic flow but reduces the overall diffusiophoretic force, potentially resulting in unstable trapping of the nanoparticle and decreased trapping stiffness (verified in Fig. 6b). And a decreasing in temperature will lead to a converse result. However, as shown in Fig. S1, temperature is directly proportional to laser power. In practice, we adopted a 785 nm laser with good stability, leading to minimal fluctuations during operation. Moreover, laboratory temperature is controlled by an air conditioner, further reducing temperature fluctuations. This systematic investigation shown here clearly confirms that CRONT can be readily used for conducting subsequently bio-molecular identification tasks.

### Optothermal trapping of proteins and DNAs

**Fig. 4:**
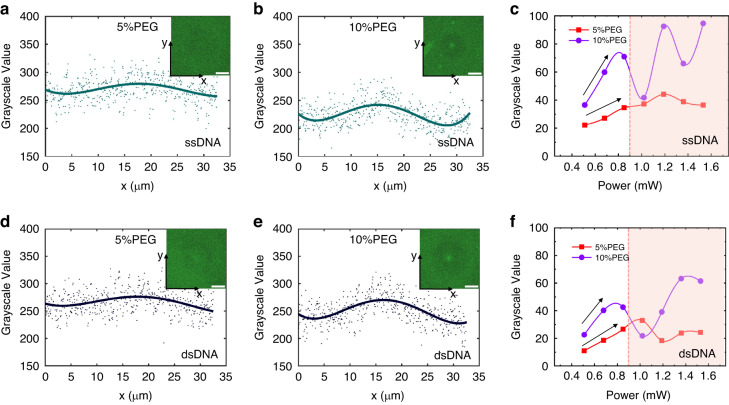
Optothermal trapping of ssDNA and dsDNA. **a**, **b** The fluorescent distribution of FITC labeled ssDNA (59 bp) in two different PEG solutions (5% and 10%). **c** The relationship between the peak-to-valley grayscale value of ssDNA aggregation and laser power at two different PEG solutions. **d**, **e** The fluorescent distribution of FITC labeled dsDNA (59 bp) in two different PEG solutions (5% and 10%). **f** The relationship between the peak-to-valley grayscale value of dsDNA aggregation and laser power at two different PEG solutions. The data points in (**a**), (**b**), (**d**), and (**e**) are the accumulation grayscale value of the fluorescence signal in the y-direction, and the solid lines are the corresponding fitted curves of the data points via least square method. The concentration of ssDNA and dsDNA are 50 μM and 25 μM respectively, the heating laser power is 0.84 mW, the scale bars = 30 μm

Furthermore, to enable CRONT, we also investigated the aggregation behaviors of proteins and DNAs via FITC labeling (FITC labeling method is shown in Supplementary Note 7). In the case of DNA, the length of rigid stem is important for the force generated by the PEG concentration gradient. For the dsDNA, the diffusiophoresis of longer base pair (>8 bp) can be accumulated in PEG (wt%>5%) solution, while for the ssDNA below 120 bp, the accumulation in PEG (wt = 5%) is not obvious^14^. And our experimental study went consistence with these former results. As illustrated in Fig. 4, when we activate the heating laser, both ssDNA and dsDNA of 59 bp exhibit good accumulation at the hotter region in PEG (wt = 10%). Faint color “band-sides” can be observed around the laser heating centers, as it shows in the insets of Fig. 4. This is a result of DNA aggregation at the laser heating center, leading to a depletion of DNAs from the surrounding “band-side” region. Moreover, as Fig. 4c, f indicate, under a certain laser power of 0.9 mW, we observed that higher laser power and larger PEG concentration result in a higher accumulation rate, which indicated by the peak-to-valley grayscale value. While higher laser power will not continually increase the accumulation rate due to the enlarged thermo-osmotic flow. Notably, the accumulation rate of ssDNA was found to be higher than that of dsDNA.

**Fig. 5:**
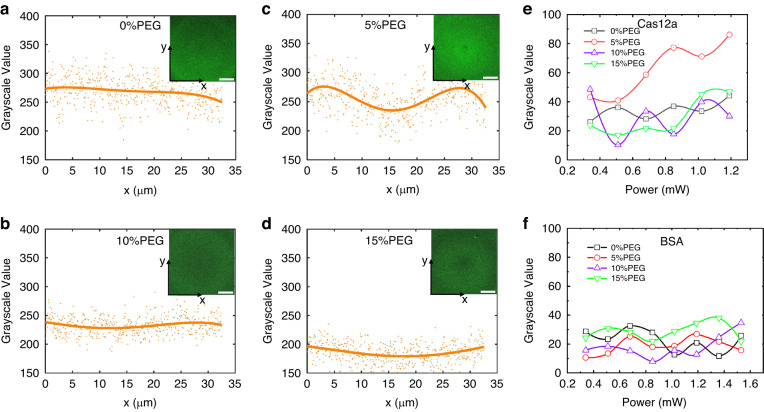
Optothermal trapping of Cas12a and BSA proteins. **a**–**d** The fluorescent distribution of FITC labeled Cas12a protein in four different PEG solutions (0–15%), the data points are the accumulation grayscale value of the fluorescence signal in the y-direction, and the solid lines are the corresponding fitted curves of the data points via least square method, the concentration of Cas12a is 28 μM (M_w _= 160 KDa), the heating laser power is 0.5 mW, the scale bars = 30 μm. **e**, **f** The relationship between the peak-to-valley grayscale value of Cas12a(**e**)/BSA(**f**) aggregation and laser power at different PEG solutions. The concentration of BSA is 18.5 μM (M_w _= 67 KDa)

Moreover, although the proteins accumulation has been rarely studied, we found that the FITC labeled Cas12a proteins exhibit a tendency to form slight ring-like accumulations (Fig. 5a–e), which indicates that the ${D}_{T}$ of Cas12a is positive while close to 0. And the increasing of the laser power results in a higher accumulation rate. However, interestingly, our results demonstrate that the variations in PEG concentration do not directly correlate with the accumulation rate. In other words, regardless of the presence or absence of PEG, the ring-like accumulations of Cas12a proteins remain similar. In addition, we also conducted tests on the commonly used protein of bovine serum albumin (BSA) proteins with FITC labeling. As depicted in Fig. 5f, align with the results in ref. ^17^, in the presence of an optothermal field, the distribution of BSA remains random and unaffected by the presence of PEG molecules, which means the ${D}_{T}$ of BSA protein is close to 0. This phenomenon can be attributed to the soft morphology and the comparable size (few nanometers) of the protein with respect to the PEG network. Hence, the direct impact of the PEG-induced diffusiophoresis force (${D}_{T}^{{diff}}$) on the protein is minimal. Instead, the intrinsic diffusion coefficient (${D}_{T}$) of the protein plays a dominate role in its optothermal accumulation behavior. And the FITC labeling procedure and more biomolecule trapping data are shown in Supplementary Notes 8, 9.

### CRONT for nucleotide identification

**Fig. 6:**
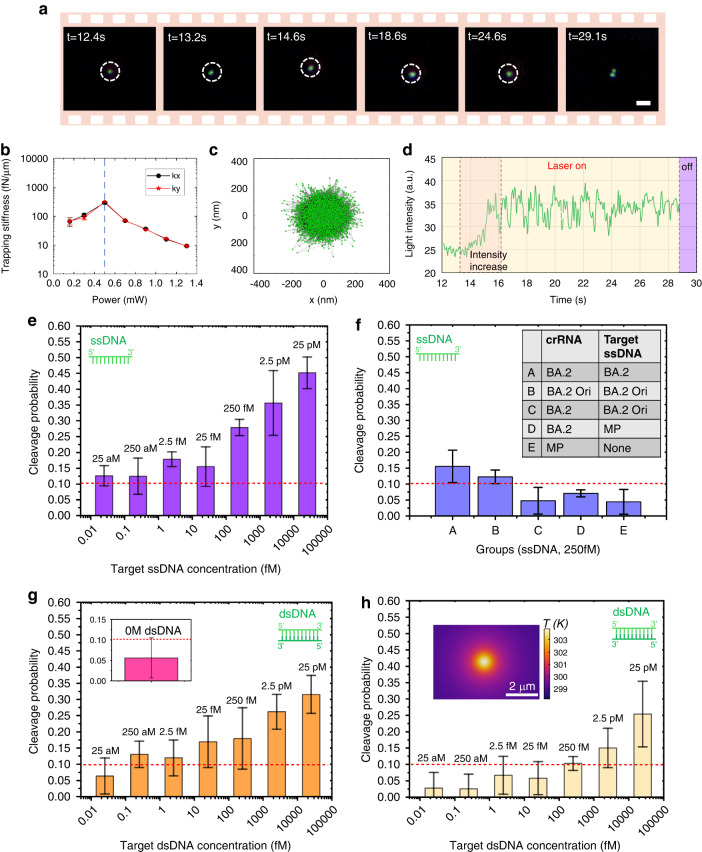
CRONT system for nucleotide detection and identification. **a** A single DNA@AuNS is captured by the CRONT at the laser heating region. The heating laser is turned off at 28.8 s, and cleavage is observed afterward. **b** Trapping stiffness measurements at varying laser powers in x/y direction, with the dashed line denoting the maximum stiffness at 0.5 mW. **c** Position distribution of the trapped single DNA@AuNS at 0.5 mW. **d** Light intensity variation of a trapped DNA@AuNS during the laser activation. The target ssDNA is from part of the Monkeypox (MP) virus sequence. Frames were recorded using dark-field microscopy, and the scale bar is 2 μm. The full video is available in Supplementary Video 3. **e** Cleavage probability of the DNA@AuNS at different target ssDNA (MP) concentrations. **f** Cleavage probability at different crRNA and target ssDNA combination groups (A-E) for specificity test, the target ssDNA concentrations is 250 fM. **g** Cleavage probability of the DNA@AuNS at different target dsDNA (MP) concentrations. The optical power set as 0.5 mW in (**a**), (**c**–**g**). **h** Cleavage probability of the DNA@AuNS under dsDNA at a lower optical power of 0.16 mW, the inset indicates the temperature distribution. Each capturing event was conducted for 2 min, and each data point comprised 10–17 capturing events over a 40-min period. Each concentration was tested three times. The corresponding sequences of crRNA, ssDNA, and dsDNA are shown in Supplementary Note 10. The PEG mass fraction is 10%. The concentration of AuNS and Cas12a is 0.5 μM and 0.125 nM respectively

The optothermal field involved in CRONT can provide a suitable temperature condition (37 °C) for the CRISPR-based bio-detection, and its capability to provide enrichment of bio-nanoparticles can enable DNA detection at ultralow concentration by replacing Brownian motion governed diffusive detection. Here, we used the CRISPR-Cas12a scheme, which has a unique ability of indiscriminate cleavage for ambient ssDNA, i.e., the trans-cleavage effect^41–43^. When CRISPR-Cas12a is activated by the target DNA, it can cleave the ssDNAs which connect the DNA@AuNS conjugate. During the operation of CRONT, a 10 μL solution containing three elements (Sol (CRONT), preparation method is shown in Supplementary Note 10), i.e., target ssDNA, Cas12a-crRNA complex, and DNA@AuNS conjugate, was injected into the microfluidic chamber. Then, by turning on the heating laser (0.5 mW), we can find and capture a single DNA@AuNS conjugate with an average diameter of 80 nm (see Supplementary Note 11). When activated by the target DNA, Cas12a-crRNA complex can cleave the DNA@AuNS conjugate at the heating laser center where the localized temperature is about 37 °C (Fig. 1c–e). We can switch off the heating laser intermittently for better examination of the cleavage of the DNA@AuNS conjugate (see Supplementary Note 12 for more detail). As Fig. 6a shows, when we switched off the laser at 28.8 s, the DNA@AuNS was found to split into two parts (positive result) and re-dispersed into the solution. Cleavage typically occurs within 2 min. For better observation of the separation and to prevent the entry of other DNA@AuNS particles, one can periodically check every 20 s by temporarily turning off the laser and repositioning it (see Supplementary Video 3, 4). As shown in Fig. 6b, c, We also quantify the trapping stiffness of a DNA@AuNS conjugate using the equipartition method^44^. The measured data reached a peak of 293 ± 14 fN·μm^−1^ at 0.5 mW. An increase in temperature amplifies the thermo-osmotic flow but reduces the diffusiophoretic force, leading to a decrease in trapping stiffness. This aligns with the simulation and analysis in Fig. 3. Moreover, as demonstrated in Fig. 6d, while trapping a single DNA@AuNS, we also observe an increase in its scattered light intensity due to cleavage. However, the more notable confirmation of cleavage comes from observing the two separated parts.

We also noticed that false results may occur in the following situations: (i) the DNA@AuNSs might aggregate without the connection of ssDNA; (ii) The target ssDNA of low concentration may not enter the potential well during the operation of CRONT. Hence, activated trans-cleavage may not be observed even if the target ssDNA exists, particularly in a lower concentration condition. Therefore, as Fig. 6b depicts, we also tested target ssDNAs (Monkeypox (MP) virus sequence) with a series of final concentrations ranging from 25 aM to 25 pM to investigate the number of cleavage events during a set of CRONT capturing events. Each capturing event (continued for 2 min) was independent, in which we manually changed the capturing locations in the microfluidic chip right after one capturing event ended. We found that a higher target DNA concentration can lead to a higher cleavage probability. And the cleavage probability of 0.1 is set as the threshold for a positive result according to the control experiments (Group E) in Fig. 6c. Hence, the limit of detection (LOD) of target ssDNA (MP) is 25 aM.

More importantly, we also tested the specificity of the CRONT approach, as shown in Fig. 6c, through conducting an experimental matrix of different crRNAs and target DNAs combinations. For example, target DNAs of S gene of SARS-CoV-2 wild-type (labeled as BA. 2 Ori) and the Omicron BA. 2 variant can only be identified by their corresponding Cas12-crRNA complex, whereas the scramble sequence or crRNA from MP virus did not generate positive signal. The results indicated that a single DNA@AuNS conjugate can only be cleaved with the right-paired target DNA-crRNA combination. Similarly, we also tested the performance for dsDNA detection. The LOD for dsDNA is 250 aM (Fig. 6d), which is higher than that of ssDNA due to the higher accumulation rate of ssDNA (Fig. 4). Furthermore, the cleavage probability decreases under lower laser power (0.16 mW) due to the reduced reaction temperature (Fig. 6e). In addition, we also conducted the fluorescence-based CRISPR-Cas12a DNA detection, which further verify the CRONT scheme (See Supplementary Note 13).

Therefore, the CRONT system exhibits DNA identification ability at single molecule level. Note that there was only a single base difference in target DNAs of S gene of SARS-CoV-2 wild-type and the Omicron BA. 2 variant within the region for crRNA identification, which indicates the identification ability of the CRONT system for SNP. With the fully illustrated sensitivity and specificity of this system, it can broadly be used to identify emerging variants of SARS-CoV-2 such as BQ.1, and XBB etc.

## Discussion

In this study, by incorporating the diffusiophoresis and thermo-osmotic flows in the boundary layer of an optothermal responsive film, we have demonstrated a novel nano-manipulation technique entitled CRONT. CRONT has enabled the immediate implementation of CRISPR-based biosensing within ultra-low detection volume. Optical tweezers are now endowed with DNA identification ability via the CRISPR-based biosensing system. The localized heating character of CRONT has provided an avenue for biomolecule enrichment and a thermal environment necessary for the cleavage of CRISPR complex. Therefore, we have remolded the classical nanotweezers into a molecular probe with exceptional specificity for SNP detection and a detection limit in attomole level.

On the other hand, the CRONT system has also improved the classic CRISPR sensing strategy, which has been limited by Brownian motion diffusion of target DNA. Further development of this optothermal-based CRISPR bio-detection scheme may rely on an array of laser heating spots for parallel high-throughput detection, which makes the technique more suitable for quantitative detection at much reduced detection time. Beyond CRISPR-based bio-detection, the biocompatibility and universality of this optothermal manipulation scheme also make it suitable for a broader range of optothermal-based bio-detection tasks. One may use CRONT to guide the CRIPSR/Cas complex to the target DNA and initiate the gene editing process at predetermined times for specific research purposes. It also allows the researchers to monitor the gene editing process in real time at single molecule level. We believe that this non-contact nanoprobe will inspire researchers to address more biological issues for next-generation nano-manipulation and point-of-care diagnostics in the future.

Despite the reaction occurring in a microscale region, the thermo-osmotic flow and diffusiophoresis consistently transport the target DNA into the reaction area. Consequently, the LOD of the CRONT is also restricted by the volume of the microfluidic chip, currently set at only 10 μL. We anticipate that a larger microfluidic sample volume would lead to a lower LOD. With further development, CRONT holds immense promise as a tool for advancing our understanding of complex biological processes and serving as a versatile detection probe in the fields of biomedical research, disease diagnosis, and drug discovery.

## Materials and methods

### Experimental setup

The optical setup was based on a Nikon inverted microscope (Ti2-E), with a 785 nm solid-state laser (OBIS 785LX, Coherent) that was expanded (5×) and reflected by a non-polarizing plate beam splitter (BS, R: T = 90:10, 700–1100 nm, Thorlabs). A 100× oil objective (Nikon, numerical aperture (NA) = 1.3) was used to focus the laser onto the optothermal substrate. Optical images were captured using a colored sCMOS camera (2048 × 2048, Dhyana400DC, Tucsen Ltd.). To block the reflected 785 nm laser, a short pass filter (SPF, OD = 4, λ_cutoff_ = 675 nm, Edmund) was utilized. Dark-field oil condenser (Nikon, NA = 1.43–1.20) and bright-field condenser were employed for sample illumination. The fluorescence excitation was performed using a mercury lamp (Nikon C-HGFI Intensilight Fiber Optic Illuminator).

### Materials preparation

To create the optothermal substrate, a thin layer of gold (10 nm) was deposited onto a coverglass measuring 20 × 20 mm. Prior to the gold film deposition, an adhesion layer of chromium (5 nm) was also applied. These deposition procedures were carried out by Suzhou Hengxin Microelectronics CO., Ltd. We also conducted measurements of transmittance (T) and reflectance (R) for a 10 nm gold film with a 5 nm Cr adhesion layer using a PerkinElmer Lambda 950 UV/Vis/NIR spectrophotometer. At λ = 785 nm, the *T* = 27%, *R* = 31.6%, and $\left(1-R-T\right)=41.4 \%$. The microfluidic chip consisted of a glass slide of 76 × 26 mm, along with a coverglass that had the Au film deposited on it. The two slides were sealed and separated by a thin parafilm layer. The resulting microfluidic chip had a height of ~30 μm.

### Finite elements method simulations

We used COMSOL Multiphysics to calculate the temperature distribution and force distribution around the optothermal substrate. A 2D-axisymmetric geometry model composed of a gold film, glass substrate, and solvent was built. The simulation utilized material parameters are obtained from the COMSOL material library. And an optothermal heat source with a Gaussian profile was set at the interface of the gold film and the solvent, which presented as:$Q\left(r,z\right)={P}_{0}(1-R-T)\frac{2\alpha }{\pi {\omega }_{0}^{2}}{e}^{-2{r}^{2}/{\omega }_{0}^{2}}{e}^{-\alpha z}$, where ${P}_{0}$ denotes the incident laser power, ${\omega }_{0}$ is the laser beam waist, the absorption coefficient *α* = 4πn^’^/λ (7.65 × 10^7 ^m^−1^), with n^’^ representing the imaginary part of the refractive index. And heat transfer and laminar flow models in fluids were used to calculate the thermophoresis and thermos-osmotic flow field. Room temperature was set as 25 °C at all other boundaries except for the optothermal heat source. In addition, to calculate the optical force, we used the electromagnetic wave model in frequency domain and dipole approximation was adopted for obtaining optical gradient force and scattering force exert on the nanoparticles^40^.

### Supplementary information

Supplementary Information for CRISPR-powered optothermal nanotweezers: Diverse bio-nanoparticle manipulation and single nucleotide identification

Supplementary Video 1

Supplementary Video 2

Supplementary Video 3

Supplementary Video 4
