# Supplementary material for: CRISPR-powered optothermal nanotweezers: Diverse bio-nanoparticle manipulation and single nucleotide identification
Source: Light Sci Appl. 2023 Nov 16;12:273. doi: 10.1038/s41377-023-01326-9 (PMC10654382; doi:10.1038/s41377-023-01326-9)
Supplement: Supplementary file 1 — Supplementary Information for CRISPR-powered optothermal nanotweezers: Diverse bio-nanoparticle manipulation and single nucleotide identification [file 41377_2023_1326_MOESM1_ESM.docx]

# Supplementary Information for “CRISPR-powered optothermal nanotweezers: Diverse bio-nanoparticle manipulation and single nucleotide identification”

Jiajie Chen^1,5^*, Zhi Chen^1,5^, Changle Meng^1,5^, Jianxing Zhou^1^, Yuhang Peng^1^, Xiaoqi Dai^1^, Jingfeng Li^1^, Yili Zhong^1^, Xiaolin Chen^1^, Wu Yuan^2^, Ho-Pui Ho^2^*, Bruce Zhi Gao^3^, Junle Qu^1^, Xueji Zhang^4^, Han Zhang^1^*, Yonghong Shao^1^*

^1^ State Key Laboratory of Radio Frequency Heterogeneous Integration, Key Laboratory of Optoelectronic Devices and Systems of Ministry of Education and Guangdong Province, College of Physics and Optoelectronics Engineering, Shenzhen University, Shenzhen 518060, China

^2^ Department of Biomedical Engineering, The Chinese University of Hong Kong, Shatin, Hong Kong, China

^3^ Department of Bioengineering and COMSET, Clemson University, Clemson, SC, 29634, USA

^4^ School of Biomedical Engineering, Shenzhen University, Shenzhen, Guangdong, 518060, China

^5^These authors contributed equally: Jiajie Chen, Zhi Chen, Changle Meng

*E-mails: [cjj@szu.edu.cn](mailto:cjj@szu.edu.cn), [aaron.ho@cuhk.edu.hk](mailto:aaron.ho@cuhk.edu.hk), [hzhang@szu.edu.cn, shaoyh@szu.edu.cn](mailto:hzhang@szu.edu.cn,%20shaoyh@szu.edu.cn).

**Supplementary Videos**

Supplementary Video 1: Trapping and releasing of DNA@AuNSs.

Supplementary Video 2: Translocation of a DNA@AuNS cluster.

Supplementary Video 3: The observation of a DNA@AuNS conjugate trapping and separation in mobile mode for DNA identification.

Supplementary Video 4: The observation of a DNA@AuNS conjugate trapping and separation in a fixed position for DNA identification.

# Supplementary Note 1

# Temperature measurement method

The temperature measurement method is based on a phase transition effect of a liquid crystal 5CB[^1^](#_ENREF_1), which undergoes a phase transition at 35 ℃[^2^](#_ENREF_2). It can measure the maximum temperature in the laser focusing region. As shown in Fig. S1, by filling the microfluidic chamber with 5CB at room temperature ($T_{0}$= 25 ℃), we observed that the radius of the phase transition region (R_5CB_) increases linearly with the incident laser power in CRONT. This relationship can be determined through a linear fit of eight measured data points at different laser powers, in our case, yielding the equation R_5CB_=5.987P-0.987. Consequently, the phase transition occurs at $P_{0}$= 0.16 mW, where R_5CB_ = 0 and the transition temperature of 35 ℃ is reached. Subsequently, the maximum temperature increment $\boldsymbol{\Delta}T_{max}^{5CB}$ at different laser powers can be calculated using the equation of$\boldsymbol{\Delta}T_{max}^{5CB}={\left( 35 ℃-T_{0} \right)P}/{P_{0}}$. Furthermore, taking into account the higher thermal conductivity of water (0.6 Wm^−1^ K^−1^) compared to 5CB (0.15 Wm^−1^ K^−1^) [^3^](#_ENREF_3), we calculated the temperature increment in water as $\boldsymbol{\Delta}T_{max}^{water}$= 0.6$\boldsymbol{\Delta}T_{max}^{5CB}$. Therefore, the maximum temperature in water solution is $T_{max}^{water}$=$T_{0}+0.6\boldsymbol{\Delta}T_{max}^{5CB}$=${T_{0}+0.6 \left( 35 ℃-T_{0} \right)P}/{P_{0}}$.

For example, at laser power of 0.5 mW, we measured that the maximum temperature is 43.75 ℃. The experimental measurements align well with our simulation results of temperature distribution in Fig.1c-d. Therefore, we can calculate the average temperature is 37 ℃ around the laser spot, which derived from the temperature distribution within a 1 μm radius semi-circular region around the laser focus center (refer to Fig. 1d in the main test).


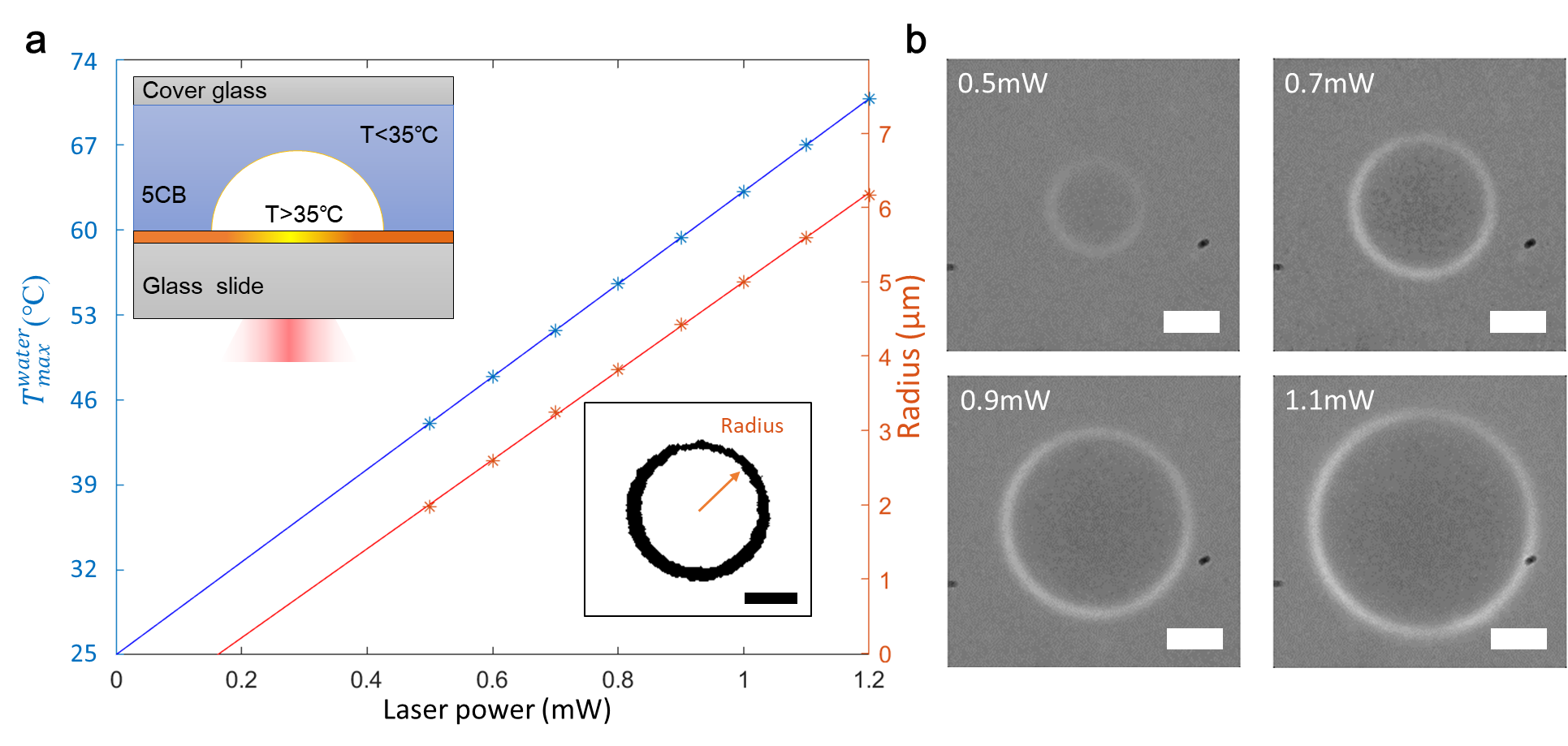


**Fig. S1 Temperature measurement method. a**, Schematic diagram illustrating the measurement method, showcasing the measured inner radius of the phase transition region in 5CB and its corresponding maximum temperature relative to the incident optical power. And graphic binarization was applied prior to radius measurement (the inset showcases an example at 0.7 mW). **b**, Dark field images of 5CB corresponding under four different incident laser powers, with scale bars indicating 3 μm.

# Supplementary Note 2

**PEG network correlation length ξ**

Typically, the concentration gradient of PEG leads to the movement of suspended particles towards the hotter region, known as the diffusiophoretic force. Specifically, PEG, being a neutral solute, accumulates in the boundary layer of the particle[^4^](#_ENREF_4), which significantly enhances its slip velocity at the particle surface. And the interaction potential $\varphi$ between solute molecules and particles will lead to an excess pressure to drive the particle’s movement. Normally, boundary layer approximation is adopted to analysis this particle-polymer boundary interaction induced diffusiophoresis[^5^](#_ENREF_5)^,^[^6^](#_ENREF_6).

This approximation is applicable to rigid particles that are considerably larger than the surrounding PEG molecules. If polymer volume fraction ($\phi$) is larger than its critical volume fraction $\phi^{*}$, the PEG molecules are within the semidilute regime, the molecules form a three-dimensional network composed of polymer chains, resulting in polymer blobs. This network can be characterized by a correlation length known as ξ, which decreases as the polymer volume fraction ($\phi$) increases (${{\xi R_{g}(\phi}^{*}/\phi)}^{0.75},$ for PEG-10000, gyration radius $R_{g}$ is 4.18 nm and $\phi^{*}$=4.7 %)[^7^](#_ENREF_7). Therefore, at PEG mass fractions of 5 % and 10 %, the correlation lengths ξ are estimated to be around 4.2 nm and 2.6 nm, respectively. Consequently, PEG molecules can be regarded as smaller fragments of the polymer network on the scale of ξ. This implies that at these concentrations diffusiophoresis can directly impact the relatively larger DNA@AuNS (Diameter of 80 nm) through particle-polymer interactions.

In addition, it should be noted that the mass fraction and volume fraction of PEG are closely related. Taking into account the density of the PEG ($\rho_{PEG}$) and solvent ($\rho_{sol}$), the mass fraction $\omega={\phi\cdot(\rho}_{PEG}/\rho_{sol})$ [^8^](#_ENREF_8). For example, at PEG mass fractions of 5% and 10%, the corresponding volume fractions (ϕ) are 4.5% and 9%, respectively.

# Supplementary Note 3

**Diffusiophoresis term calculation**

To calculate$D_{T}^{diff}=\frac{k_{B}}{3\eta}R_{g}^{2}n\left( TS_{T}^{PEG}-1 \right)$, in our case, the molecule weight ($M_{w}$) of PEG is 10 kg·mol^−1^, the $n=cN_{A}$, where $N_{A}$ is the Avogadro constant. at mass fraction of 5%, viscosity $\eta$ is 2.57 ×10^-3^ Pa🞝s[^8^](#_ENREF_8), PEG gyration radius $R_{g}=0.02{M_{w}}^{0.58}$=4.18 nm [^9^](#_ENREF_9), and $S_{T}^{PEG}$ is 0.064 K^−1^ [^10^](#_ENREF_10). We can calculate $D_{T}^{diff}$= 2.0 µm^2^ K^−1^ s^−1^ which is larger than the thermophoretic mobility of some commonly used metal nanoparticles ($D_{T}$≈ 0.1 µm^2^ K^−1^s^−1^) [^11^](#_ENREF_11). Therefore, the $D_{T}-D_{T}^{diff}$<0, which means the gold nanoparticle can be driven to the laser heating center.

# Supplementary Note 4

**Thermo-osmotic flow calculation**

The thermo-osmotic coefficient $\chi$ in Equation 4 denotes all interfacial interactions at the surface under a temperature gradient, which is described by DLVO theory[^12^](#_ENREF_12). The DLVO theory suggests that the interaction potential comprises both repulsive electrostatic and attractive van der Waals contributions (vdW). Consequently, the DLVO potential is specific to the materials involved and can be described by the surface's zeta potential (ζ) and the material system's Hamaker constant ($A_{H}$). The macroscopic $A_{H}$ quantifies the interaction between material 1 and material 2 mediated by an inter-medium material 3[^13^](#_ENREF_13).

Therefore, the thermo-osmotic coefficient$\chi$, is primarily influenced by the contributions of the electric double layer ($\chi_{E}$) and van der Waals ($\chi_{vdW}$) interactions. By applying the Debye-Hückel approximation, we can express this relationship as follows:

$\chi=\chi_{E}+\chi_{vdW}=\frac{\varepsilon\zeta^{2}}{8\eta}+\frac{A_{H}\beta T}{3\pi\eta d_{0}}$ (S1)

Due to the uneven distribution of PEG molecules around the hotter center (described by Equation 2), we utilize the viscosity value ($\eta$=2.5723 ×10^-3^ Pa🞝s) at 5 % PEG to calculate the trapping forces in 10 % PEG. This accounts for the depletion of PEG molecules from the center of the laser spot, where the temperature rises by approximately 10 K. Additionally, we employ β = 0.2 × 10^−3^ K^−1^ as the thermal expansion coefficient of water, and $d_{0}=$0.2 nm as the cut-off parameter [^6^](#_ENREF_6).

For the electrostatic contribution, we adopted a zeta potential of ζ = −30 mV for gold film[^14^](#_ENREF_14), and a static permittivity of ε = 80 ε_0_ is utilized for the dielectric constant of water. Regarding the Hamaker constant, $A_{H}$, for the interaction between AuNS in the presence of a water medium, it is generally observed that the Hamaker constant for metals is larger than that of dielectric materials. For the Au-water-Au system, a typical value is around 25·10^−20^ J, while a thin Au film on a glass surface exhibits a lower range of 1×10^−20^-6 ×10^−20^ J[^14^](#_ENREF_14). In our calculations, we used a value of $A_{H}$ = 3.3·10^−20^ J for simplicity. Therefore, by considering the combined contributions, the thermo-osmotic coefficient of AuNS is determined as χ_Au_ = 3.9 × 10^−10^ m^2^·s^−1^.

# Supplementary Note 5

**Optical force calculation**


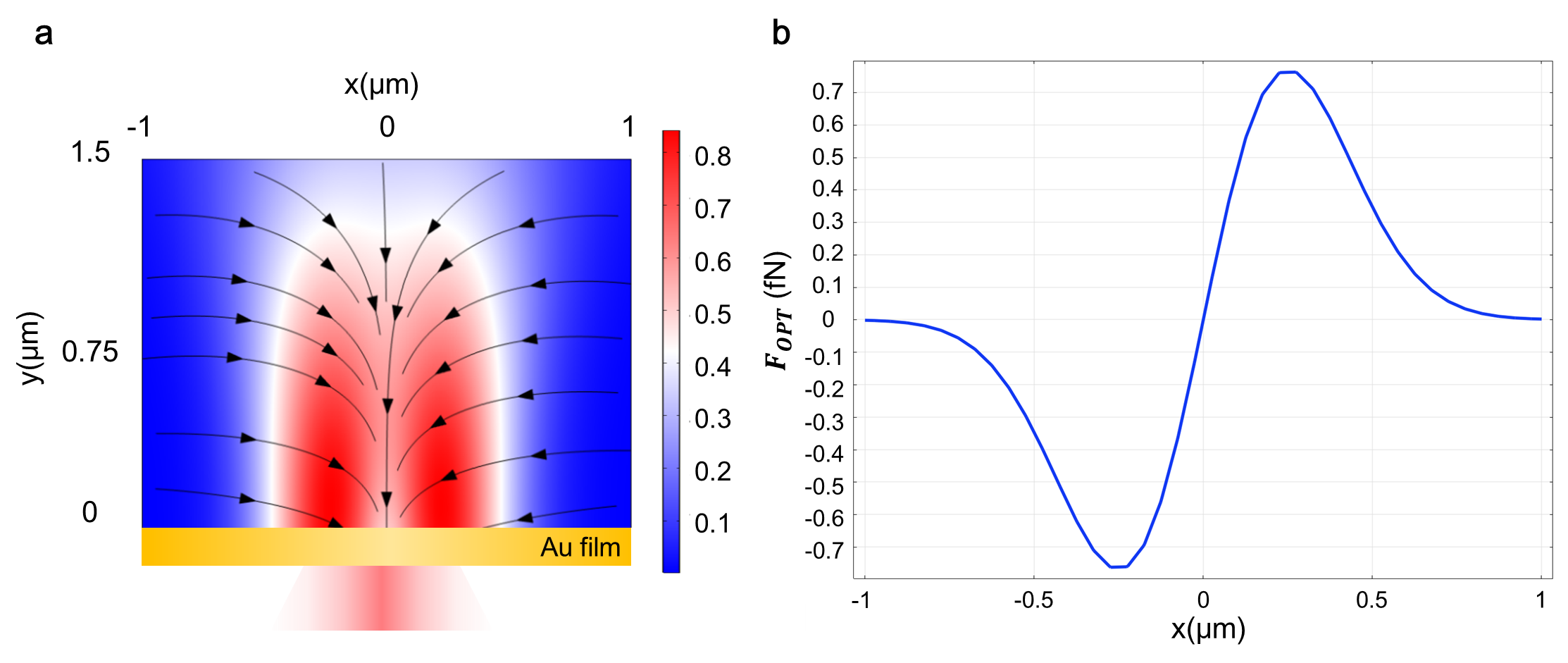


**Fig. S2 Optical force calculation. a,** The distribution of optical forces experienced by a gold particle of 80 nm in diameter near the focal spot, with the gold film located at y = 0, and the beam propagating along the positive y-axis direction. **b,** The lateral optical force distribution experienced by the 80 nm gold particle positioned approximately 100 nm above the gold film. The incident laser power is 0.5 mW.

We computed the optical forces, taking into account the complex polarizability correction term[^15^](#_ENREF_15)^,^[^16^](#_ENREF_16). Prior to the calculation, we also measured that the transmission coefficient (T) of the Au film is 27 % at the wavelength of 785 nm. Consequently, as illustrated in Fig. S2, for the optical force simulation at an incident laser power of 0.5 mW, we employed a laser power of 0.5 mW multiplied by 27 %. The beam's waist radius is 500 nm. This analysis reveals that the optical force ($\boldsymbol{F}_{\boldsymbol{OPT}}$) is approximately one order of magnitude lower compared to the predominant thermodynamic forces ($\boldsymbol{F}_{\boldsymbol{D}}$**,** $\boldsymbol{F}_{\boldsymbol{TO}}$) in this trapping configuration (as shown in Fig. 3h).

**Supplementary Note 6**

**Temperature field homogenization** **correction factor calculation**

When dealing with metal particles, their high thermal conductivity creates a uniform temperature gradient field that minimizes temperature differences around the particles, thus decreasing their thermophoretic velocity. And we can define $n=\frac{\kappa_{p}}{\kappa_{s}}$ as the ratio of the thermal conductivity of the particle ($\kappa_{p}$) to thermal conductivity of the surrounding solution ($\kappa_{s}$). For instance, under the same temperature gradient field, metal particles with higher thermal conductivity (n>1) exhibit a lower thermophoresis velocity of $u_{m}$. While for the same sized dielectric particles (Polystyrene beads, virus, proteins, or DNAs) with similar thermal conductivity to the surrounding solution (n≈1), they exhibit a higher thermophoresis velocity of $u_{d}$, which are unsusceptible to the temperature field homogenization. To conveniently calculate the thermophoresis velocity of the metal nanoparticles. One can employ a simple correction factor in the form of thermophoresis velocity ratio which can be written as[^17^](#_ENREF_17):

$\frac{u_{m}}{u_{d}}=1+\frac{1-n}{2+n}\frac{6\frac{R^{2}}{R_{g}^{2}}\left( \ln\left( 1+\frac{R_{g}}{R} \right)-\frac{1}{1+\frac{R}{R_{g}}} \right)}{3+2\frac{R_{g}}{R}}$ (S2)

In our case, the thermal conductivity of Au is $\kappa_{Au}=518$W·mK^-1^, and it will become smaller as DNA@AuNS is composite of 15nm AuNS and DNA, from Ref. [^18^](#_ENREF_18), we can estimate the thermal conductive of DNA@AuNS is $\kappa_{p}$= 150 W·mK^-1^. While the thermal conductivity of solution is $\kappa_{s}$= 0.6 W·mK^-1^. Therefore, in that case, the thermal conductivity ratio $n=250$ [^19^](#_ENREF_19), from Eq. S2, we can obtain ${u_{m}}/{u_{d}}\approx0.2012$. Subsequently, this correction factor is used in our simulations.

# Supplementary Note 7

**FITC labeled Cas12a protein synthesis**

The Cas12a protein was dissolved in 0.1 M sodium carbonate buffer (pH 9) following ultrafiltration, while FITC was dissolved in anhydrous DMSO. The Cas12a protein solution and FITC were mixed at a molar ratio of 3:1 (FITC/protein). The reaction solution was incubated at 4 ℃ for 8 hours, followed by the addition of 50 mM NH_4_Cl to stop the reaction, and then incubated for an additional 2 hours at 4 ℃. After ultrafiltration, the product was dissolved in PBS buffer. Prior to further experimentation, the FITC-labeled Cas12a protein was stored at -20 ℃.

**FITC labeled dsDNA synthesis**

The dsDNA template was prepared according to the Annealing Oligonucleotides Protocol by Sigma-Aldrich.

An ssDNA template of MP virus labeled with 6-FAM fluorescence at the 5’ end (5’-6-FAM-TACATTCGATAGGAACGACGAACCACCAGAGGATGATGAATAAAAAAATGATAAAATAA-3’) and its complementary strand were used. The oligonucleotides were diluted to the desired concentrations in annealing buffer containing 10 mM Tris (pH 7.5 - 8.0), 50 mM NaCl, and 1 mM EDTA. Equal volumes of the oligonucleotides were mixed in a PCR tube and heated to 95 ℃ for 2 min. The reaction mixture was then cooled to 25 ℃ over 45 min. The dsDNA template was stored at 4 ℃ until use.

# Supplementary Note 8

**Optothermal trapping of ssDNA and dsDNA**


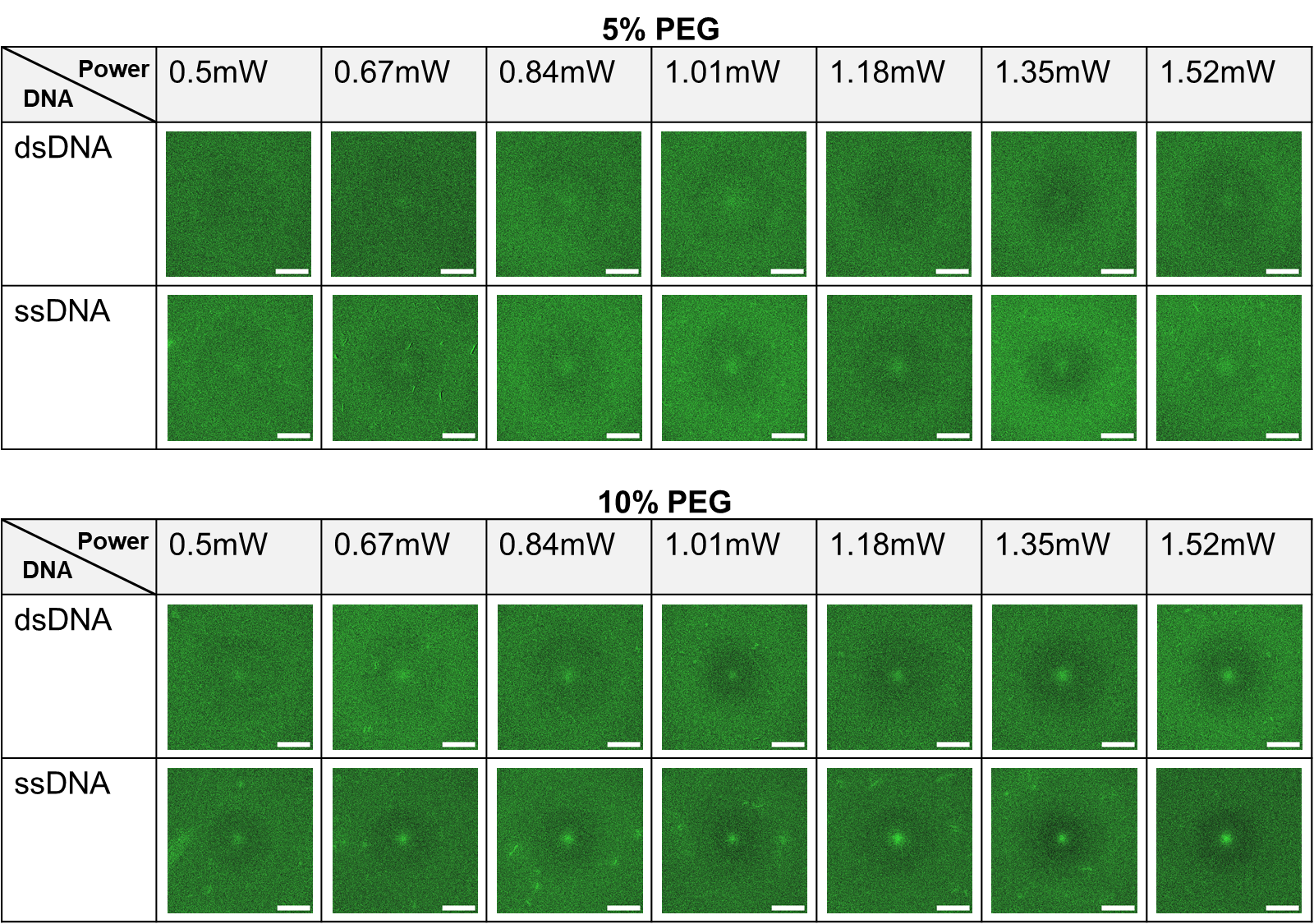


**Fig. S3** **Optothermal trapping of ssDNA and dsDNA.** The fluorescent distribution of FITC labeled ssDNA and dsDNA (59 bp) in two different PEG solutions (mass fraction 5 % and 10 %) under different laser powers. The concentration of ssDNA and dsDNA are 50 μM and 25 μM respectively, the scale bars=30 μm.

Fig. S3 shows the 2D image of fluorescent distribution of FITC labeled ssDNA and dsDNA in two different PEG solutions. In addition, to magnify and compare the molecule aggregation signal, as shown in Fig. S4-S7, we obtained data points by summing the grayscale values of the fluorescence signal in the y-direction within a length scale of approximately 30 μm in the x-direction. These data points were then fitted using the least squares method to generate corresponding curves.


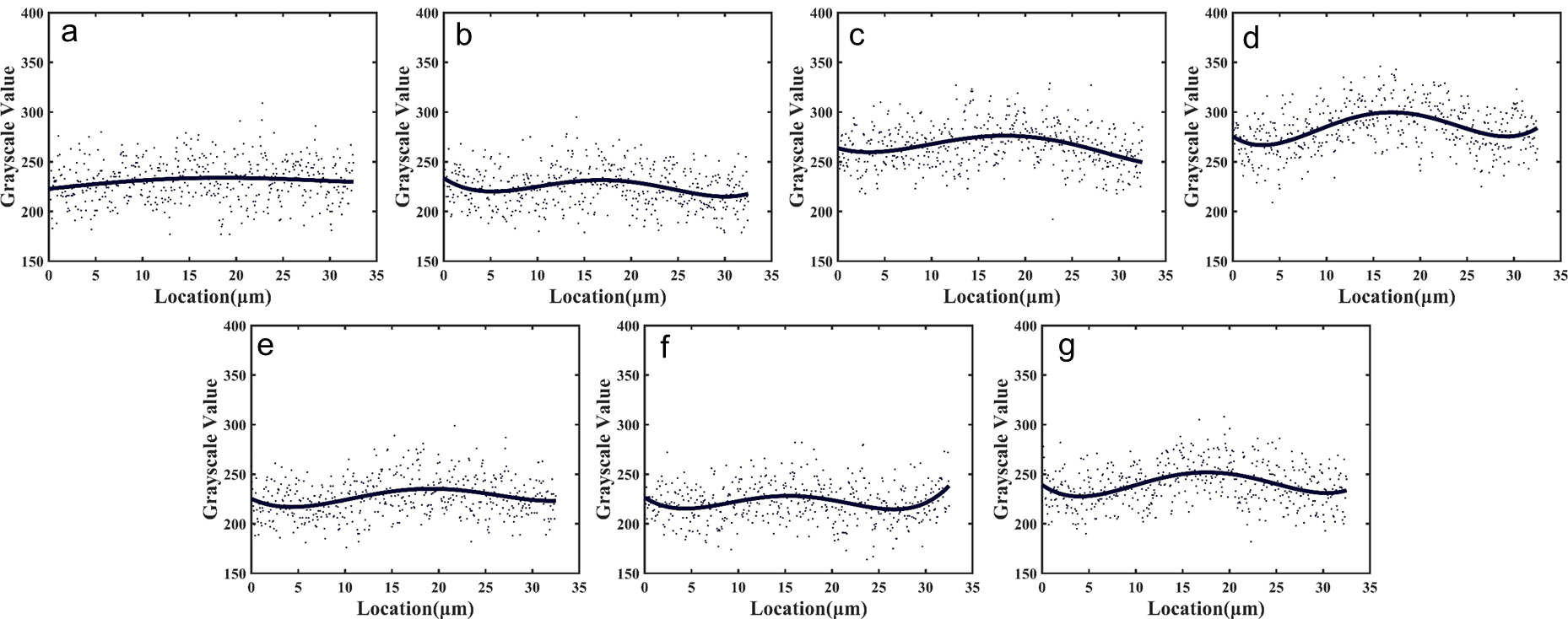


**Fig. S4 The grayscale value of the fluorescence signal for dsDNA trapping under 5 % PEG. a-g,** The grayscale curve of the dsDNA at the optical power of 0.5 mW, 0.67 mW, 0.84 mW, 1.01 mW, 1.18 mW, 1.35 mW, and 1.52 mW respectively.


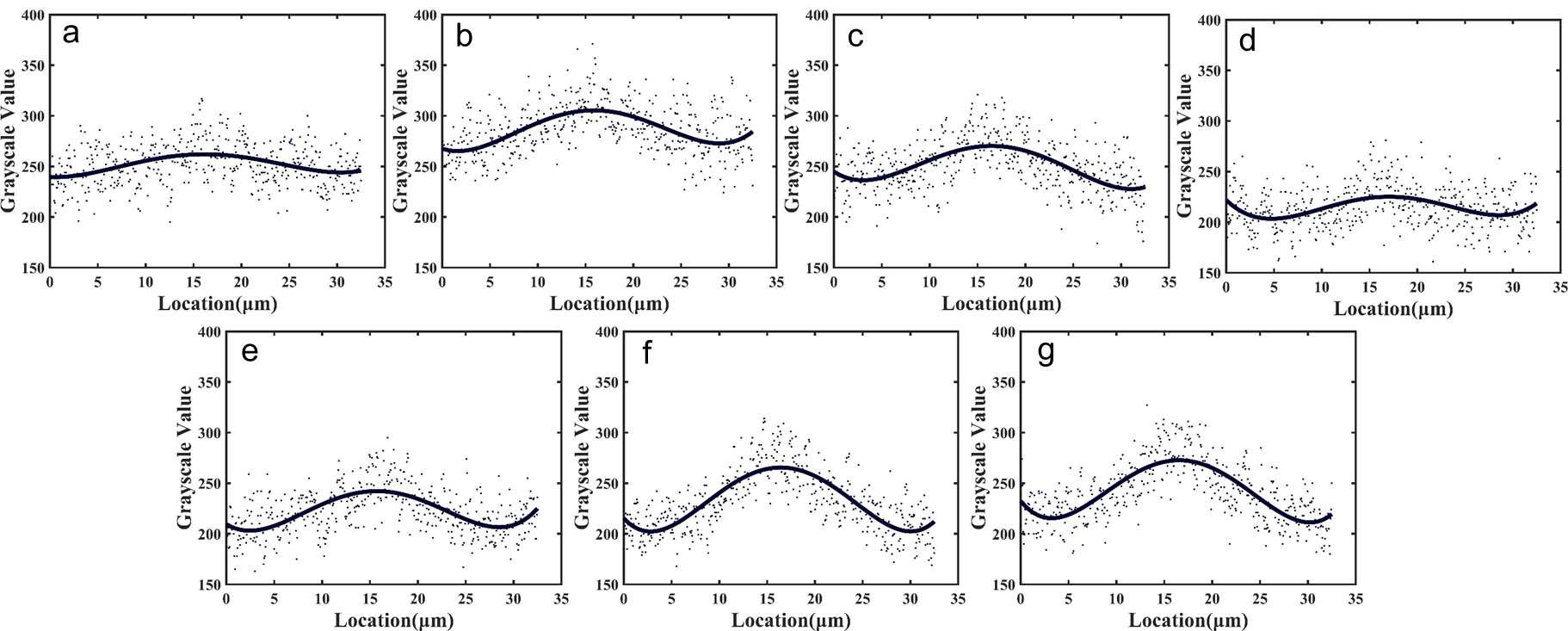


**Fig. S5 The grayscale value of the fluorescence signal for dsDNA trapping under 10%PEG. a-g,** The grayscale curve of the dsDNA at the optical power of 0.5 mW, 0.67 mW, 0.84 mW, 1.01 mW, 1.18 mW, 1.35 mW, and 1.52 mW respectively.


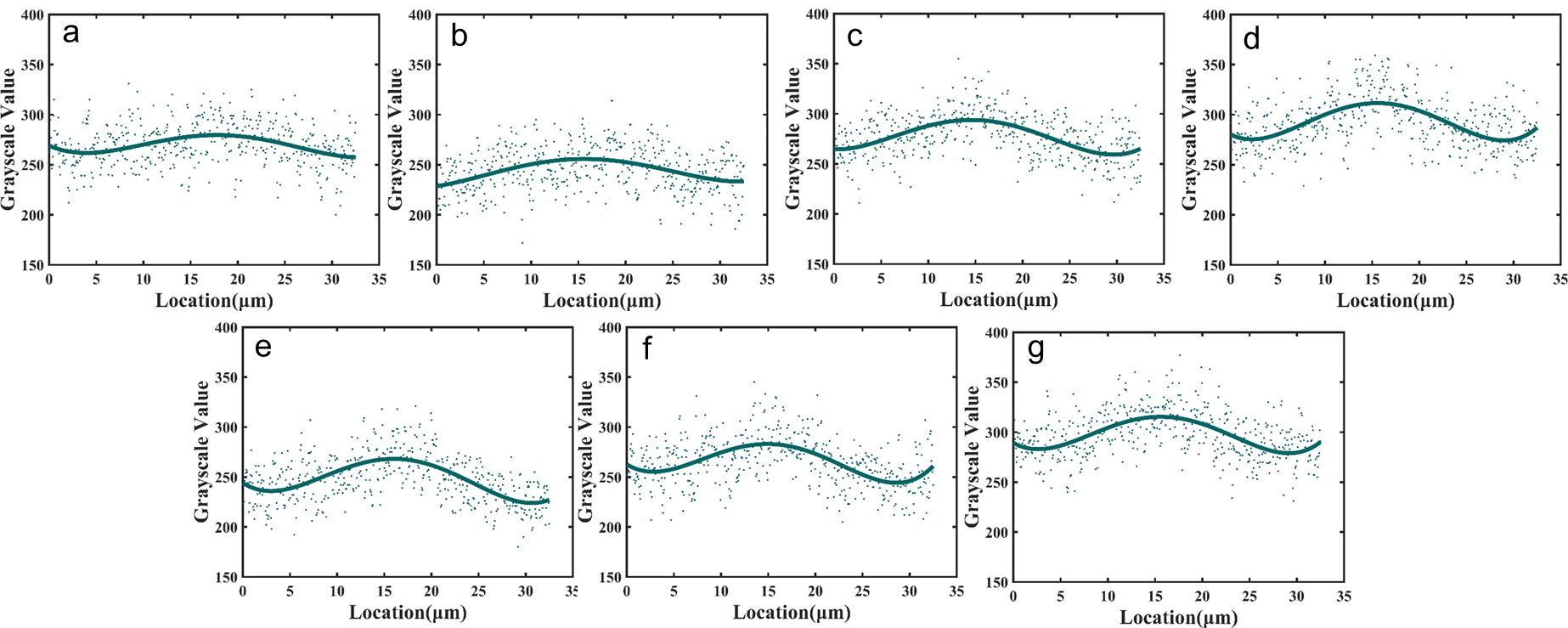


**Fig. S6 The grayscale value of the fluorescence signal for ssDNA trapping under 5%PEG. a-g,** The grayscale curve of the dsDNA at the optical power of 0.5 mW, 0.67 mW, 0.84 mW, 1.01 mW, 1.18 mW, 1.35 mW, and 1.52 mW respectively.


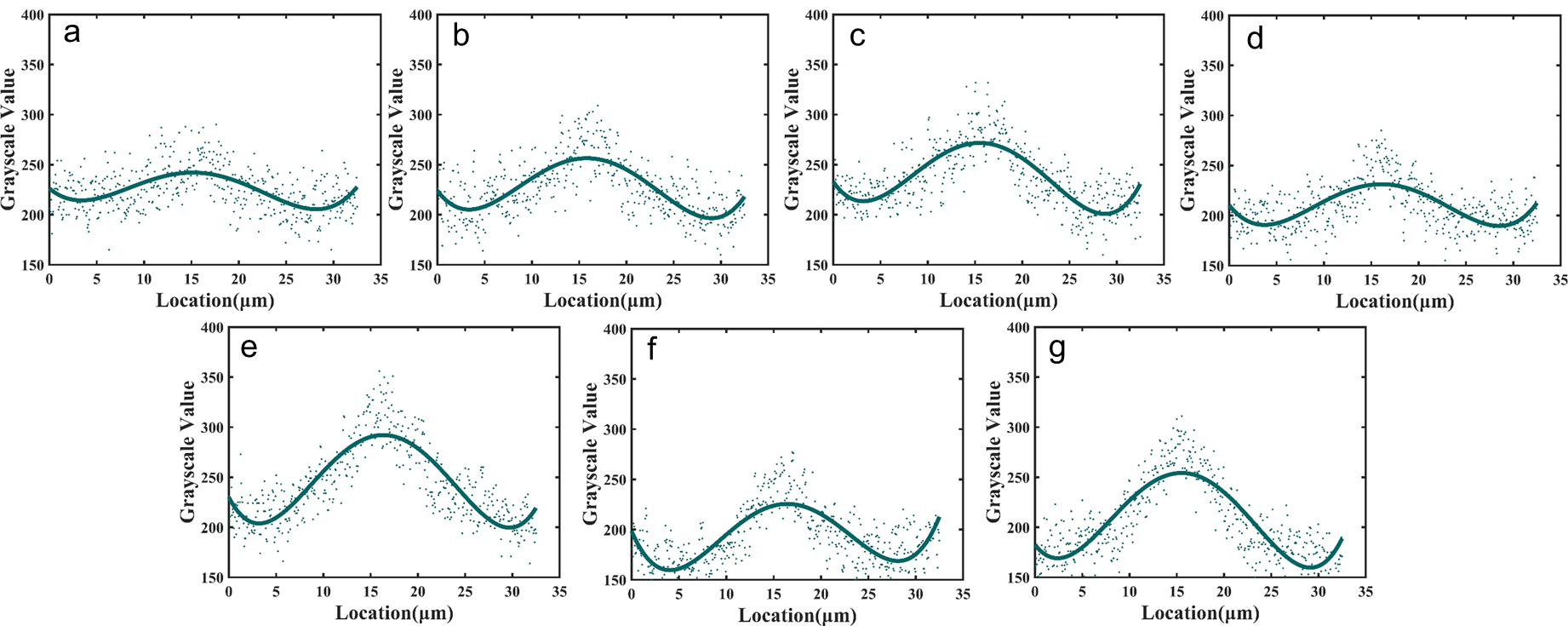


**Fig. S7 The grayscale value of the fluorescence signal for ssDNA trapping under 10%PEG. a-g,** The grayscale curve of the dsDNA at the optical power of 0.5 mW, 0.67 mW, 0.84 mW, 1.01 mW, 1.18 mW, 1.35 mW, and 1.52 mW respectively.

# Supplementary Note 9

**Optothermal trapping of Cas12a**


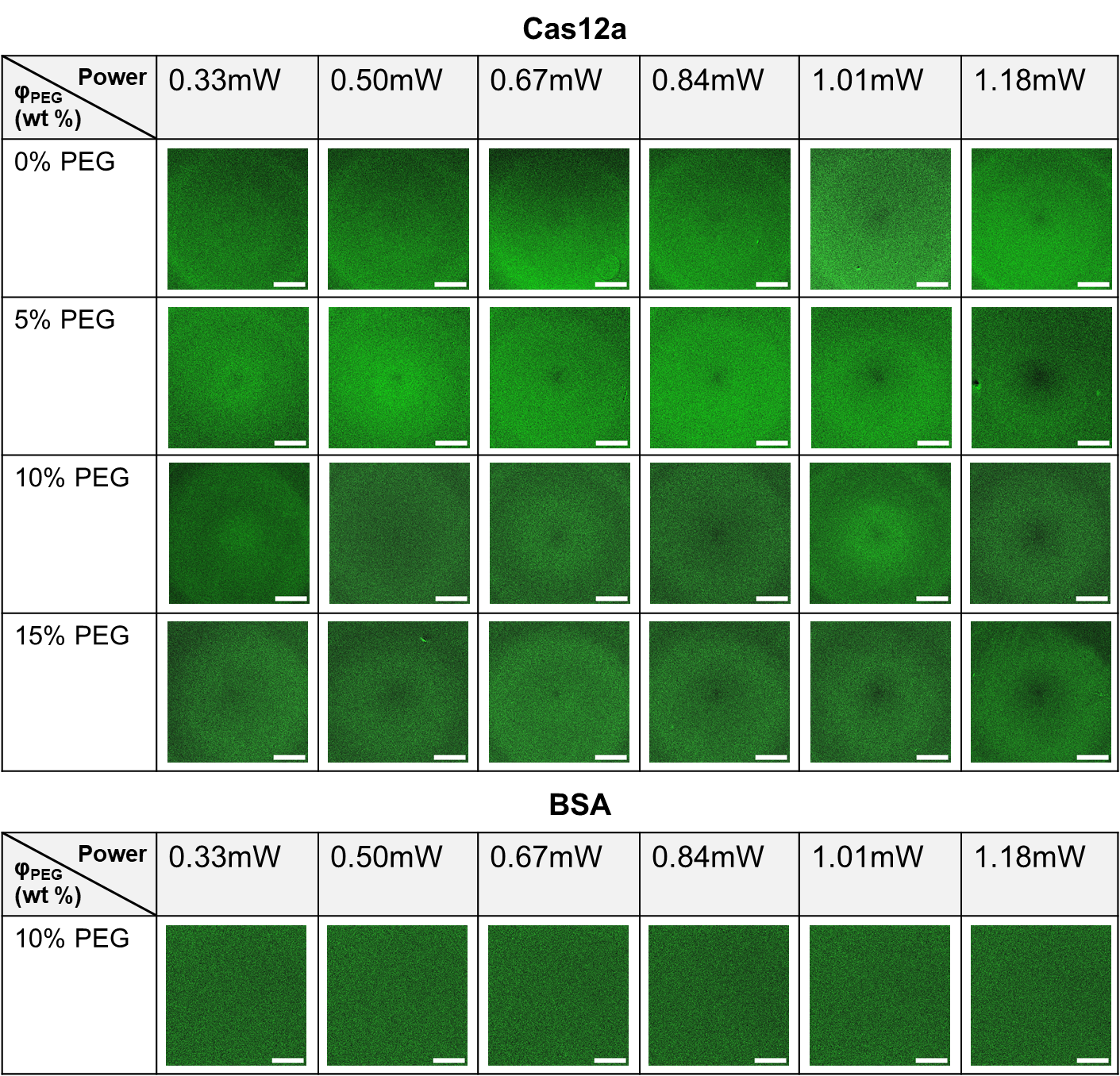


**Fig. S8 Optothermal trapping of Cas12a and BSA proteins.** The fluorescent distribution of FITC labeled Cas12a protein in four different PEG solutions (mass fraction 0%-15%) under different laser powers, the concentration of Cas12a is 28 μM (M_w_=160KDa). And the fluorescent distribution of FITC labeled BSA protein in PEG solutions of 10% under different laser powers, the concentration of BSA is 18.5 μM (M_w_=67 KDa). The scale bars=30 μm.

Fig. S8 shows the 2D image of fluorescent distribution of FITC labeled Cas12a and BSA proteins in different PEG solutions. Similar to the DNA aggregation analysis, in order to magnify and compare the proteins aggregation signal, as shown in Fig. S9-S12, we also obtained data points by summing the grayscale values of the fluorescence signal in the y-direction within a length scale of approximately 30 μm in the x-direction. These data points were then fitted using the least squares method to generate corresponding curves.


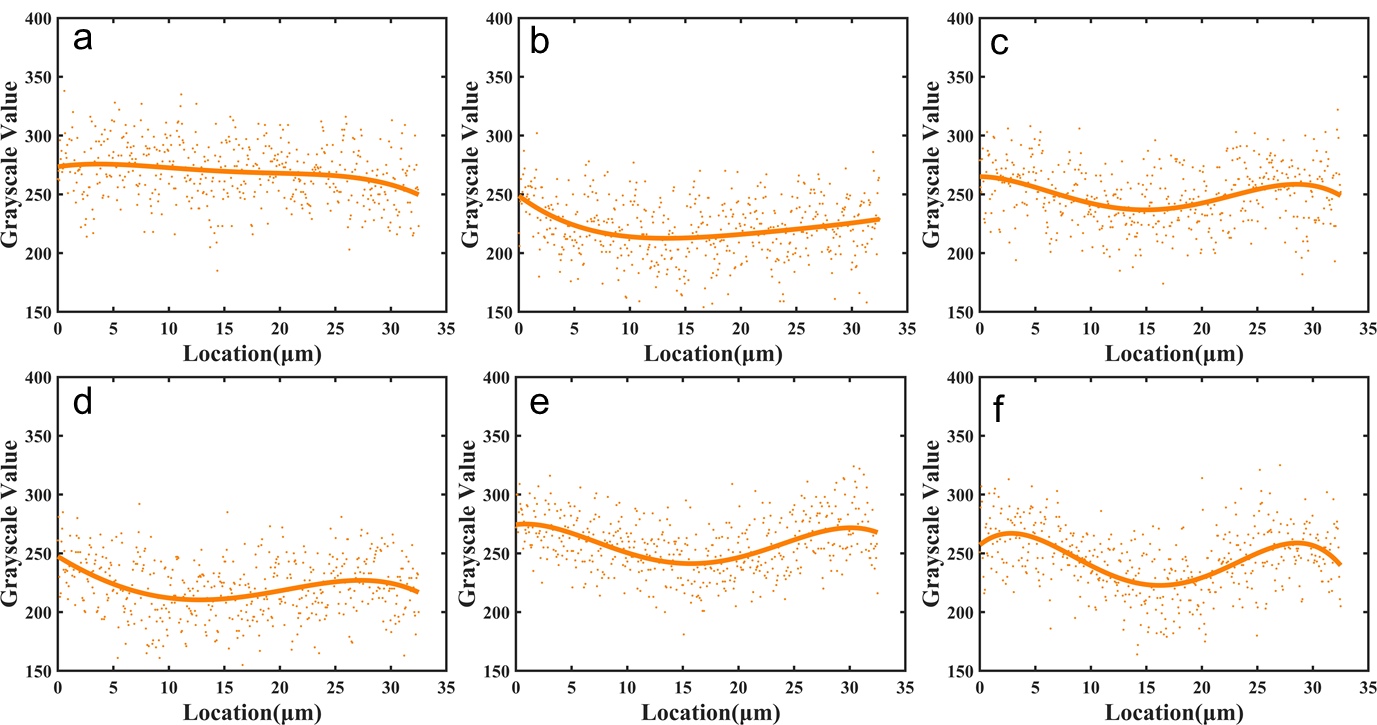


**Fig. S9 The grayscale value of the fluorescence signal for Cas12a trapping under 0%PEG. a-g,** The grayscale curve of the dsDNA at the optical power of 0.33 mW, 0.5 mW, 0.67 mW, 0.84 mW, 1.01 mW, and 1.18 mW respectively.


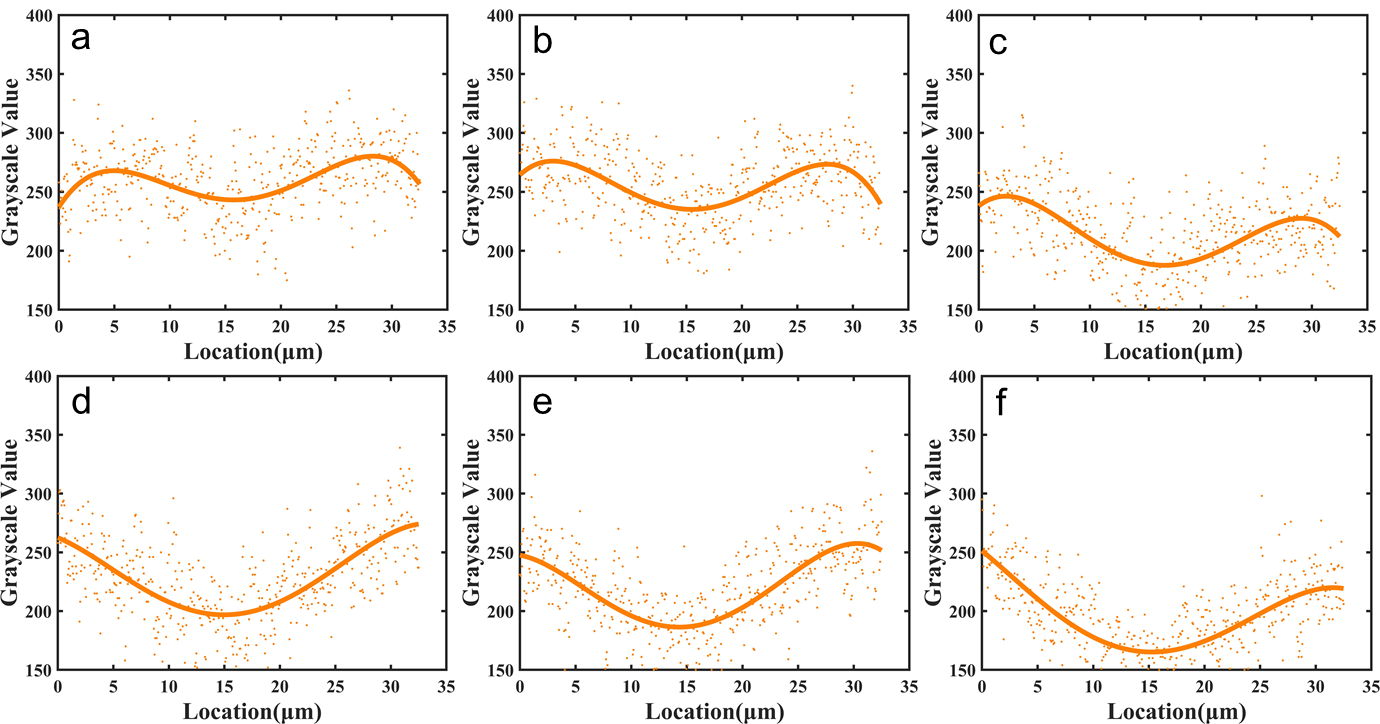


**Fig. S10 The grayscale value of the fluorescence signal for Cas12a trapping under 5%PEG. a-g,** The grayscale curve of the dsDNA at the optical power of 0.33 mW, 0.5 mW, 0.67 mW, 0.84 mW, 1.01 mW, and 1.18 mW respectively.


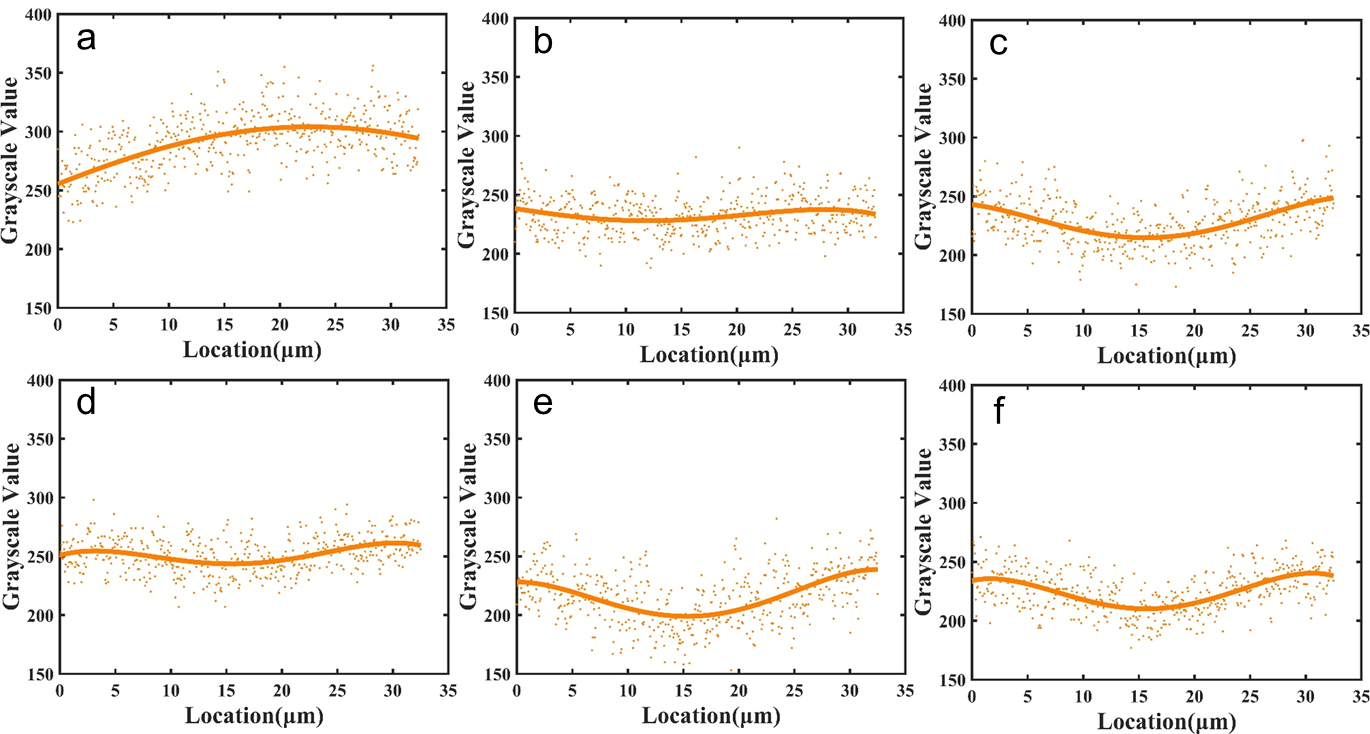


**Fig. S11 The grayscale value of the fluorescence signal for Cas12a trapping under 10%PEG. a-g,** The grayscale curve of the dsDNA at the optical power of 0.33 mW, 0.5 mW, 0.67 mW, 0.84 mW, 1.01 mW, and 1.18 mW respectively.


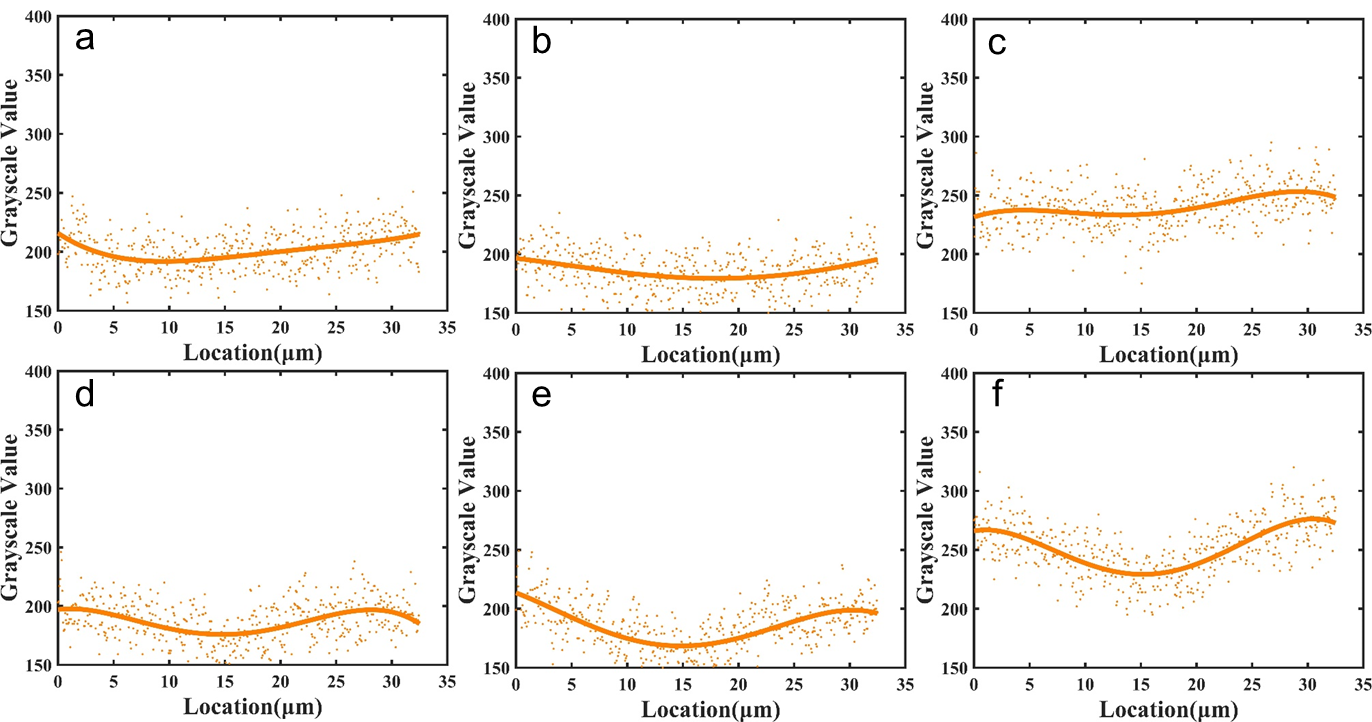


**Fig. S12 The grayscale value of the fluorescence signal for Cas12a trapping under 15%PEG. a-g,** The grayscale curve of the dsDNA at the optical power of 0.33 mW, 0.5 mW, 0.67 mW, 0.84 mW, 1.01 mW, and 1.18 mW respectively.

# Supplementary Note 10

**CRISPR/Cas12a complex preparation**

The CRISPR/Cas12a complex includes crRNA and Cas nuclease protein. The crRNA comprises an anchor sequence (UAAUUUCUACUAAGUGUAGAU for LbCas12a protein) that binds to the LbCas12a protein and a guide sequence that is identical to the target nucleic acid sequence followed by a "TTTV" PAM sequence (in case of detecting dsDNA), or inversely complementary to the target nucleic acid sequence (in case of detecting ssDNA). The target DNAs are ssDNA and dsDNA (59 bp) sequences from parts of the S gene of SARS-Cov-2 Omicron BA. 2 variant (BA.2) and wild-type (BA. 2 Ori) after the reverse-transcription, or Monkeypox (MP) virus gene sequence. Their corresponding guide sequence of the crRNA is shown in **Table S1.**

**Table S1: The sequence of CRISPR/Cas12a crRNA and target sequence combination.** Mutation sites between SARS-Cov-2 Omicron BA. 2 variant (BA.2) and wild-type (BA.2 Ori) are labeled in red, and the single nucleotide mutations within the crRNA identification region are underlined.

|  | **Target sequence (5'to3')** | **crRNA guide sequence (5'to3')** |
| --- | --- | --- |
| **MP** | TACATTCGATAGGAACGACGAACCACCAGAGGATGATGAATAAAAAAATGATAAAATAA | UAAUUUCUACUAAGUGUAGAU-UUCAUCAUCCUCUGGUGGUU |
| **BA.2** | GTGACCAACACCATAAGTGGGTCGGAAACCATATGATCGTAAAGGAAAGTAACAATTAAA | UAAUUUCUACUAAGUGUAGAU-CGAGCAUAUGGUUUCCGACC |
| **BA. 2 Ori** | GTAACCAACACCATTAGTGGGTTGGAAACCATATGATTGTAAAGGAAAGTAACAATTAAA | UAAUUUCUACUAAGUGUAGAU-CAAGCAUAUGGUUUCCAACC |

When the target nucleic acid activates the CRISPR/Cas12a complex, the CRISPR-Cas12a scheme will produce an indiscriminate cleavage for ambient ssDNA. Therefore, the DNA@AuNS conjugate which connected by ssDNA can be cut into two or more pieces due to the break of the ssDNA connection with the AuNS (see Fig. S13).

The experimental method in the experiment includes the following steps:

1. Configure CRISPR/Cas12a complex (100 nM) and dilute in reaction buffer (1× NEB 2.1);
2. First, 1μL target ssDNA\dsDNA solution (10 nM) was added into 100μL CRISPR/Cas12a complex;
3. Then, 100μL CRISPR/Cas12a complex solution was mixed with 100 μL DNA@AuNS conjugate solution (preparation method is shown in Supplementary Note 11), namely Sol (Initial);
4. Lastly, we added 1μL Sol (Initial) into 199 μL PEG-10000 solution (w/w= 10 %), which is the solution for the subsequent testing in CRONT, namely Sol (CRONT). And the final concentration of target DNA in Sol (CRONT) is 25 pM.

Note that the buffer solutions for the target DNA and DNA@AuNS conjugate are nuclease-free water. To prepare different target DNA concentrations, the 1 μL target DNA solution (10 nM) in Step 2 is diluted. Additionally, all the biological samples, such as CRISPR/Cas12a complex, target DNA, and ssDNA, are ordered from General Biol Co., Ltd.

# Supplementary Note 11

**DNA@AuNS conjugate preparation**


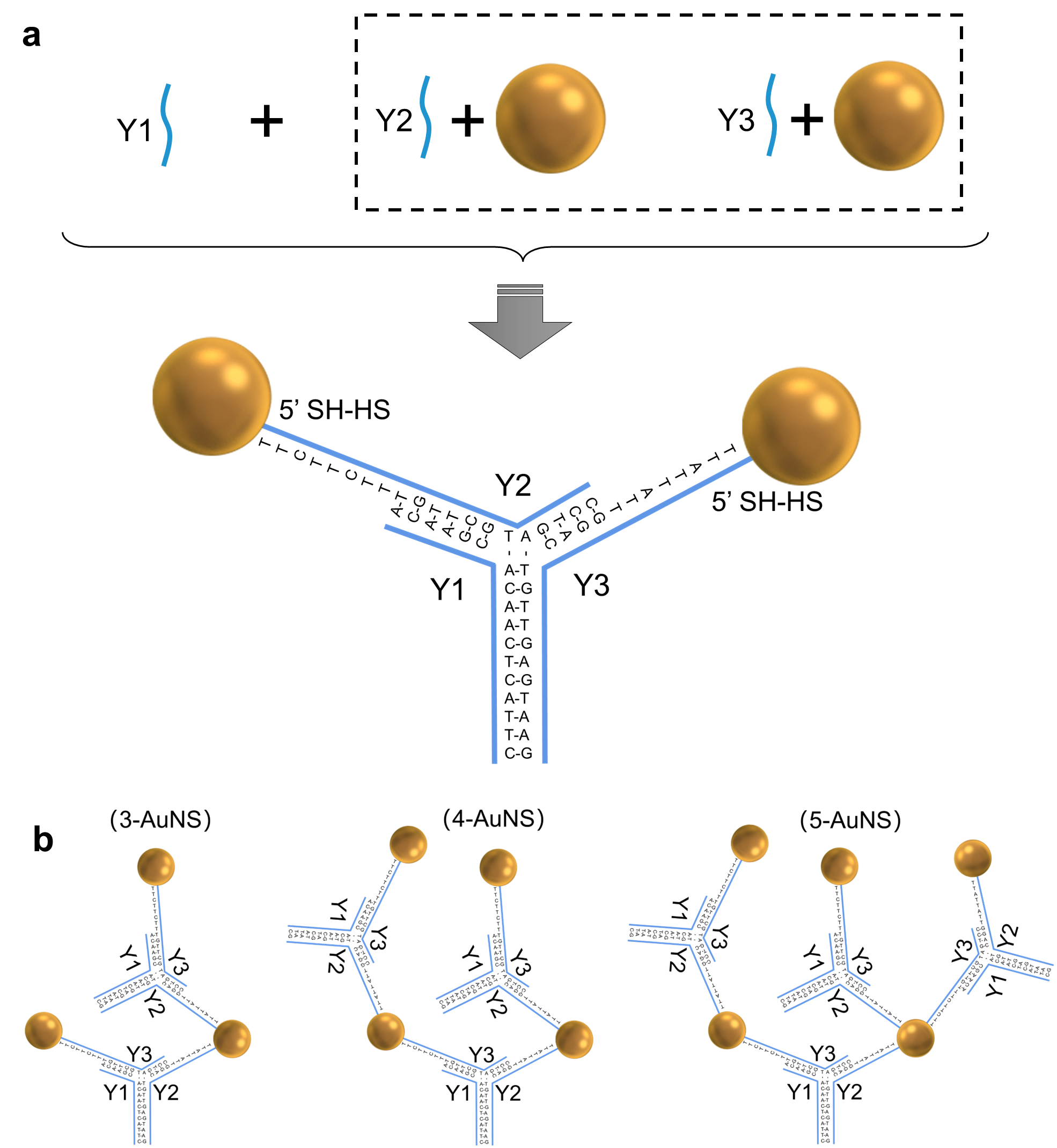


**Fig. S13 DNA@AuNS conjugate. a,** The basic structure and fabrication procedure of DNA@AuNS conjugate. **b,** Schematic of DNA@AuNS conjugates that contains multiple AuNSs, such as 3-AuNS, 4-AuNS, 5-AuNS, and so on.

Fig. S13a shows the schematic of nucleic acid AuNS conjugate preparation procedure, it is based on a "freezing and thawing" method, the detail can be found in Ref. [^20^](#_ENREF_20). We adopted a "Y-shaped" DNA@AuNS conjugate for its stable structure[^21^](#_ENREF_21). In the experiment, the Y1, Y2, and Y3 sequences are partly inversely complement to each other. The base number of Y1, Y2, and Y3 is 17, 20, and 23, respectively. The CRISPR/Cas12a complex will cut the ssDNA-connected region in the DNA@AuNS conjugate after activation by the target sequence.

As shown in Fig. S13a, the designed sequence of single-strand DNA for AuNS conjugate connection in the experiment is presented as follows:

The sequence of Y1 is 5’-CTTACTCAAACACGAACA-3’;

The sequence of Y2 is 5’-TTCTTCTTTGTTCGTAGTCC-3’;

The sequence of Y3 is 5’-TTATTATTGGACTGTTGAGTAAG-3’.

Briefly, Y2 and Y3 are linked to 15 nm AuNS to form the DNA@AuNS conjugate (Y2@AuNPs/Y3@AuNPs), while Y1 plays a role in stabilizing and strengthening the coupling structure. The production procedure of the DNA@AuNS conjugate is as follows:

1. The ssDNA of Y2 or Y3 was modified with a disulfide bond at the 5' end by the manufacturer. The disulfide bond of Y2/Y3 was reduced to the sulfhydryl group through the administration of TCEP before use.
2. Subsequently, Y2/Y3 forms a coupling compound with the AuNS (Y2@AuNS/ Y3@AuNS) through a typical freezing and thawing method [^20^](#_ENREF_20);
3. Then, the Y2@AuNS and Y3@AuNS solution were mixed and stirred (450 rpm) at 4 ℃. Finally, Y1 was added into the solution, and the solution was stirred (450 rpm) at 4 ℃ again to produce the final DNA@AuNS conjugate.

In addition, because one AuNS-15 nm may connect several ssDNAs on its surface, a single DNA@AuNS conjugate may contain multiple 15 nm AuNSs (see Fig. S13b). After the preparation, we measured the size and the structure of the DNA@AuNS conjugate. As its TEM image in Fig. S14 indicates, we estimated the average number of AuNSs in one conjugate, which is about 45. In addition, we also measured the average particle diameter of the DNA@AuNS conjugate via an optical nanoparticle size analyzer (Zetasizer Pro, Malvern Panalytical Ltd), which shows the diameter is 80±10 nm.


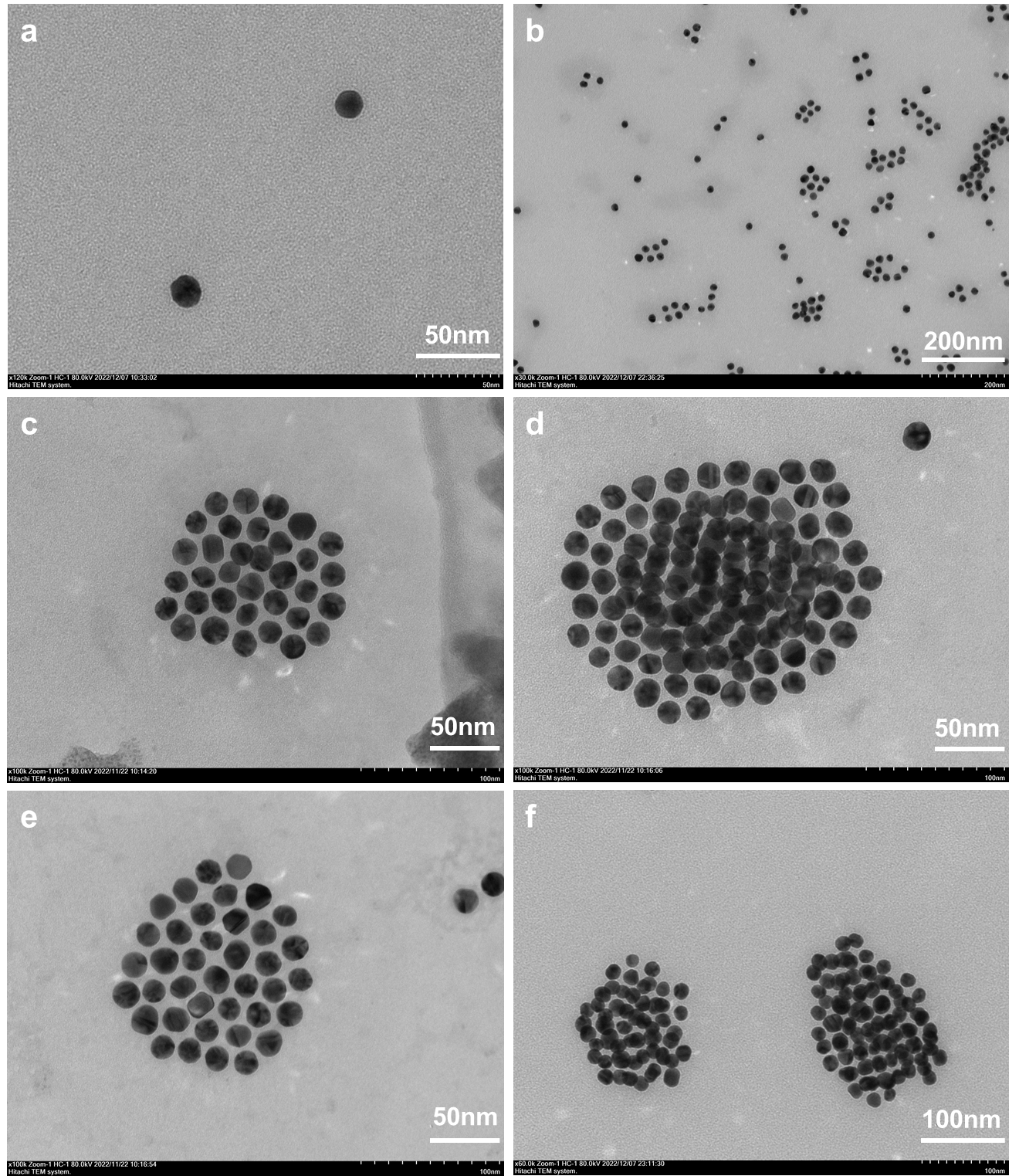


**Fig. S14 TEM raw images of** **some typical 15 nm-AuNS and DNA@AuNS conjugates. a,** TEM image of two single 15 nm-AuNSs. **b,** a broad view of some small DNA@AuNS conjugates. **c-f,** TEM image of five major DNA@AuNS conjugates in the CRONT experiments. The number of 15nm-AuNS of c-e are 34, 113, and 39 respectively, the number in **f** are 53 (left) and 79 (right).

# Supplementary Note 12

**DNA@AuNS cleavage detection**

During the CRONT-based DNA identification experiment, once a DNA@AuNS conjugate was captured at the laser focus center, cleavage may occur within 2 minutes. However, because of the optothermal net force, the separated conjugates remained together, and there was still one Airy spot in the dark-field image due to the resolution limit of the objective lens. Although the scattered light intensity slightly increased upon conjugate separation (See Supplementary Video 3), it is not direct evidence of the cleavage. To better distinguish the separated conjugates, we switched off the heating laser intermittently. The two separated DNA@AuNS conjugates disperse in the solution due to Brownian motion, leading to the observation of two Airy spots, which is a more compelling indication of the cleavage event.

Moreover, the CRONT system offers extensive flexibility in manipulation, allowing manual adjustment of the trapping positions to capture surrounding DNA@AuNS conjugates across each individual test. Fig. S15 illustrates the schematic diagram of the interrogation strategy employed in CRONT. During operation, the position can be switched between different tests to prevent recapturing of previously captured particles.

Furthermore, the thermodynamic net force consistently guides newly surrounding DNA@AuNS conjugates towards the laser focused center from the horizontal plane, as depicted in Fig. 3. This region falls within the focus depth of the objective lens, facilitating easy distinction of the captured particles. To prevent the entry of additional DNA@AuNSs during the test, one can manually adjust the laser position as demonstrated in Supplementary Video 3, showcasing the mobile mode of CRONT. In a broader context, one can capture a single DNA@AuNS conjugate at a fixed position (Supplementary Video 4) for observing the cleavage event.


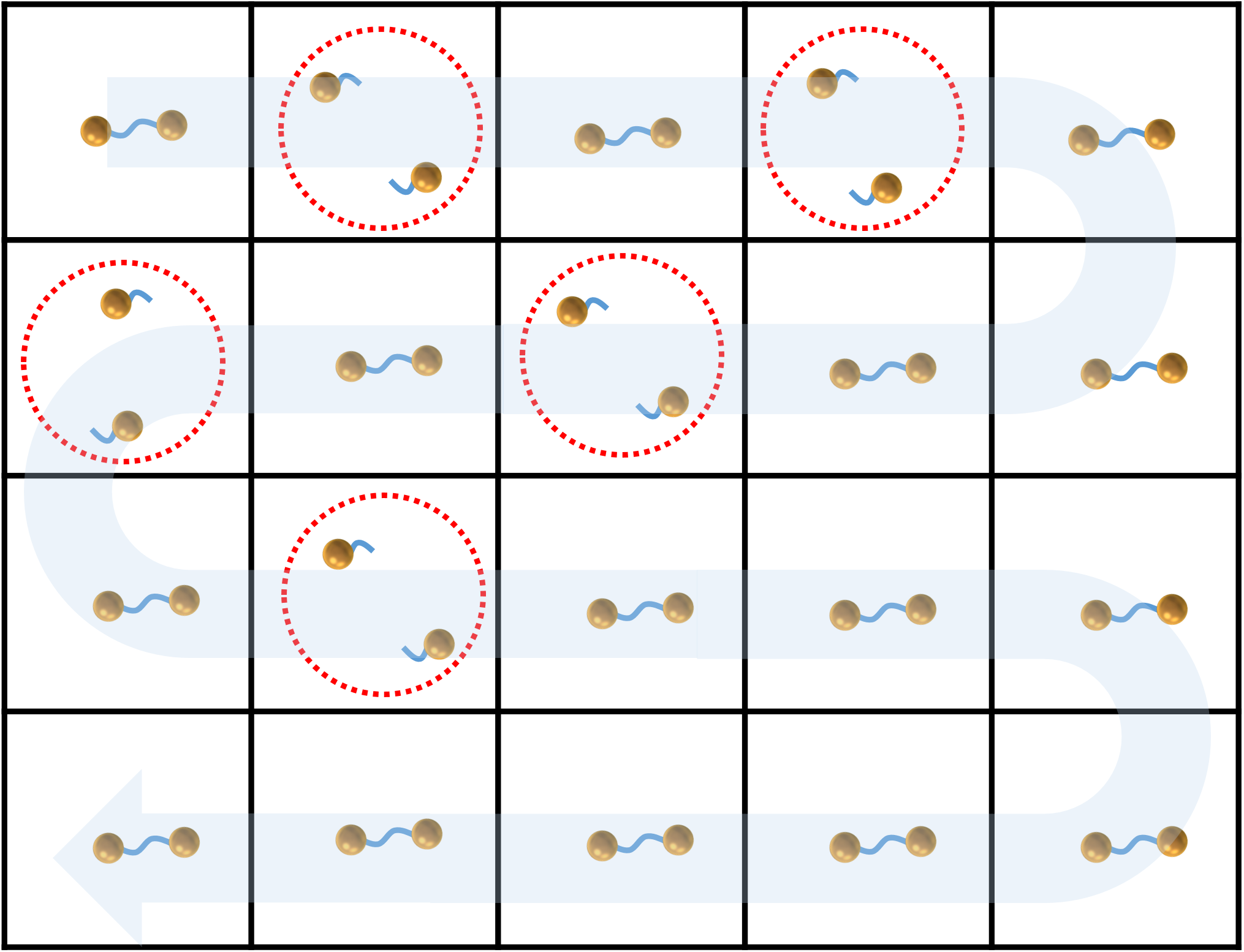


**Fig. S15 Schematic diagram of the interrogation strategy in CRONT.** The grids indicate the interrogation regions in one experiment, corresponding to an area of 133 μm ×133 μm (field of view of the CMOS), and the volume of a single interrogation region is about 0.5×10^-3^ μL. The red dashed circle denotes the cleavage of a DNA@AuNS.

# Supplementary Note 13

**Fluorescence-based CRISPR-Cas12a diagnosis verification**

As shown in Fig. S16, the successful trans-cleavage of Cas12a was validated using a fluorescence-based CRISPR-Cas12a diagnosis assay. The reaction mixture was composed of 50 nM Cas12a (NEB), 50 nM crRNA, 1×Cas12a reaction buffer, 500 nM of ssDNA reporter (5’ 6-FAM/TTATT/BHQ-1 3’, Sangon), and ssDNA or dsDNA templates. The mixture was incubated at 37 °C for 30 min and the fluorescence was under ultra-violet.


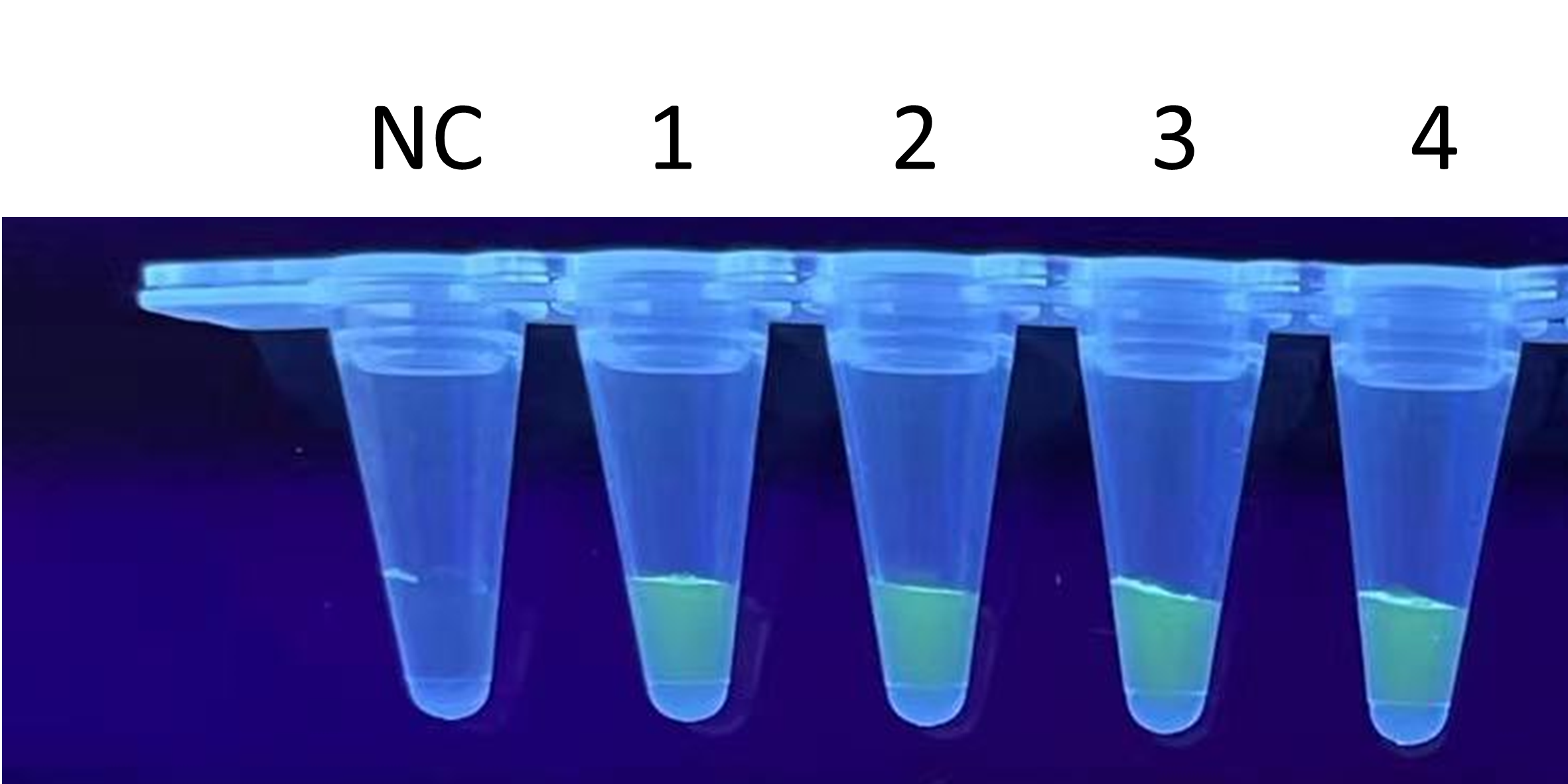


**Fig. S16 Validation of successful trans-cleavage of Cas12a with different DNA templates.** NC: no DNA template, 1: ssDNA template of MP, 2: ssDNA template of BA.2, 3: ssDNA template of BA.2 ori, 4: dsDNA template of MP.

**References**

1 Fränzl, M. & Cichos, F. Hydrodynamic manipulation of nano-objects by optically induced thermo-osmotic flows. *Nature Communications* **13**, 656 (2022).

2 Horn, R. G. Refractive indices and order parameters of two liquid crystals. *Journal de physique* **39**, 105-109 (1978).

3 Marinelli, M., Mercuri, F., Zammit, U. & Scudieri, F. Thermal conductivity and thermal diffusivity of the cyanobiphenyl (n CB) homologous series. *Physical Review E* **58**, 5860 (1998).

4 Anderson, J. L. COLLOID TRANSPORT BY INTERFACIAL FORCES. *Annu. Rev. Fluid Mech.* **21**, 61-99 (1989).

5 Anderson, J. L. Colloid transport by interfacial forces. *Annual review of fluid mechanics* **21**, 61-99 (1989).

6 Würger, A. Thermal non-equilibrium transport in colloids. *Reports on Progress in Physics* **73**, 126601 (2010).

7 De Gennes, P.-G. & Gennes, P.-G. *Scaling concepts in polymer physics*. (Cornell university press, 1979).

8 Mohsen-Nia, M., Modarress, H. & Rasa, H. Measurement and modeling of density, kinematic viscosity, and refractive index for poly (ethylene glycol) aqueous solution at different temperatures. *Journal of Chemical & Engineering Data* **50**, 1662-1666 (2005).

9 Devanand, K. & Selser, J. Asymptotic behavior and long-range interactions in aqueous solutions of poly (ethylene oxide). *Macromolecules* **24**, 5943-5947 (1991).

10 Maeda, Y. T., Buguin, A. & Libchaber, A. Thermal separation: interplay between the Soret effect and entropic force gradient. *Physical review letters* **107**, 038301 (2011).

11 Fränzl, M. & Cichos, F. Hydrodynamic manipulation of nano-objects by optically induced thermo-osmotic flows. *Nature communications* **13**, 1-9 (2022).

12 Derjaguin, B. V., Churaev, N. V., Muller, V. M. & Kisin, V. *Surface forces*. (Springer, 1987).

13 Israelachvili, J. N. *Intermolecular and surface forces*. (Academic press, 2011).

14 Giesbers, M., Kleijn, J. M. & Stuart, M. A. C. The electrical double layer on gold probed by electrokinetic and surface force measurements. *Journal of colloid and interface science* **248**, 88-95 (2002).

15 Urban, A. S. *et al.* Optical trapping and manipulation of plasmonic nanoparticles: fundamentals, applications, and perspectives. *Nanoscale* **6**, 4458-4474 (2014).

16 Kuwata, H., Tamaru, H., Esumi, K. & Miyano, K. Resonant light scattering from metal nanoparticles: Practical analysis beyond Rayleigh approximation. *Applied physics letters* **83**, 4625-4627 (2003).

17 Giddings, J. C., Shinudu, P. M. & Semenov, S. N. Thermophoresis of metal particles in a liquid. *J. Colloid Interface Sci.* **176**, 454-458, doi:10.1006/jcis.1995.9946 (1995).

18 Kodama, T., Jain, A. & Goodson, K. E. Heat conduction through a DNA− gold composite. *Nano letters* **9**, 2005-2009 (2009).

19 Franzl, M. & Cichos, F. Hydrodynamic manipulation of nano-objects by optically induced thermo-osmotic flows. *Nature Communications* **13**, 9, doi:10.1038/s41467-022-28212-z (2022).

20 Liu, B. & Liu, J. Freezing directed construction of bio/nano interfaces: reagentless conjugation, denser spherical nucleic acids, and better nanoflares. *Journal of the American Chemical Society* **139**, 9471-9474 (2017).

21 Kong, D. *et al.* Direct SARS-CoV-2 nucleic acid detection by Y-shaped DNA dual-probe transistor assay. *Journal of the American Chemical Society* **143**, 17004-17014 (2021).
